# A high-fat diet alters genome-wide DNA methylation and gene expression in SM/J mice

**DOI:** 10.1186/s12864-018-5327-0

**Published:** 2018-12-07

**Authors:** Madeline Rose Keleher, Rabab Zaidi, Lauren Hicks, Shyam Shah, Xiaoyun Xing, Daofeng Li, Ting Wang, James M. Cheverud

**Affiliations:** 1 Department of Evolution, Ecology, and Population Biology, Washington University in St. Louis, St. Louis, MO 63105 USA; 2 Department of Biology, Loyola University, Chicago, IL 60660 USA; 3 Department of Genetics, Washington University in St. Louis, St. Louis, MO 63110 USA; 4 Biology Department, 1032 W. Sheridan Road, Chicago, IL 60660 USA

**Keywords:** Obesity, Mice, Epigenetics, Methylation, RNA-seq, Gene expression, Diet

## Abstract

**Background:**

While the genetics of obesity has been well defined, the epigenetics of obesity is poorly understood. Here, we used a genome-wide approach to identify genes with differences in both DNA methylation and expression associated with a high-fat diet in mice.

**Results:**

We weaned genetically identical Small (SM/J) mice onto a high-fat or low-fat diet and measured their weights weekly, tested their glucose and insulin tolerance, assessed serum biomarkers, and weighed their organs at necropsy. We measured liver gene expression with RNA-seq (using 21 total libraries, each pooled with 2 mice of the same sex and diet) and DNA methylation with MRE-seq and MeDIP-seq (using 8 total libraries, each pooled with 4 mice of the same sex and diet). There were 4356 genes with expression differences associated with diet, with 184 genes exhibiting a sex-by-diet interaction. Dietary fat dysregulated several pathways, including those involved in cytokine-cytokine receptor interaction, chemokine signaling, and oxidative phosphorylation. Over 7000 genes had differentially methylated regions associated with diet, which occurred in regulatory regions more often than expected by chance. Only 5–10% of differentially methylated regions occurred in differentially expressed genes, however this was more often than expected by chance (*p* = 2.2 × 10^− 8^).

**Conclusions:**

Discovering the gene expression and methylation changes associated with a high-fat diet can help to identify new targets for epigenetic therapies and inform about the physiological changes in obesity. Here, we identified numerous genes with altered expression and methylation that are promising candidates for further study.

**Electronic supplementary material:**

The online version of this article (10.1186/s12864-018-5327-0) contains supplementary material, which is available to authorized users.

## Background

In the US, 35% of children are overweight and another 26.4% have obesity [1]. Obesity early in life raises the risk of obesity [2] and liver disease [3] later in life. Today 35% of adults in the United States are obese, and 42% are predicted to be by 2030 [4–6]. This is a major threat to public health, since obesity is associated with cancer, stroke, asthma, type 2 diabetes, hypertension, heart attack, and other serious health conditions [7]. The best studied causes of obesity are genetics, the environment, and their interaction [8–13]. The environment changes the expression of genes via epigenetic factors such as histone modifications, noncoding RNAs, and DNA methylation, and thus environmental factors causing obesity may do so by inducing epigenetic modifications that change gene expression. Epigenetic variation between individuals may hold the key to more accurate predictions of obesity risk, and better understanding it could lead to new tools for fighting obesity [14, 15].

Health problems can result from dysregulated gene expression. While much research has focused on the genetic variants underlying disrupted gene expression in obesity [12, 16], far less is known about how diet changes gene expression through epigenetics to cause obesity. Technological advances have made epigenetic studies more feasible, and new journals and scientific meetings have been created to address the explosion of epigenetics research [17]. The best-characterized epigenetic mechanism is DNA methylation; however, even this is not well-understood. When the majority of the cytosines in a promoter region are methylated, gene expression tends to be lower than when these regions are hypomethylated [18]. This is not always the case, however, and methylation at other regulatory regions can actually increase expression [19]. To fill in the gaps of our understanding of epigenetics, it is important to explore the methylation profile of not just promoter regions in candidate genes but of the entire genome, as we do here.

Changes in DNA methylation do not always imply changes in gene expression, or vice versa. For instance, rats fed a diet high in fat and sugar had higher hepatic expression of the *Hadhb* gene, but they had no corresponding changes in methylation [20]. Furthermore, when Rönn et al. [21] analyzed DNA methylation in men’s adipose tissue before and after 6 months of exercise, they found methylation changes in 7663 genes, but only 197 of those genes also had expression changes. This illustrates a common finding in obesity epigenetics studies: while there are methylation differences associated with obesity, many changes in DNA methylation do not cause detectable changes in the expression of nearby genes. More research needs to be done to characterize the relevant DNA methylation changes in obesity. So far, candidate-gene studies have revealed DNA methylation differences between obese and lean individuals in a handful of genes in different tissues [22–25]. Genome-wide methylation studies have revealed differentially methylated regions in genes involved in cell differentiation, the immune system, and transcriptional regulation [23]. To understand how changes in DNA methylation affect gene expression in obesity, however, it is important to consider genetic background. C57BL/6 is a widely studied mouse strain that has contributed enormously to our understanding of obesity, however its response to a high-fat diet differs from that of other mouse strains. Compared to C57BL/6, when fed a high-fat diet BALB/c mice have been shown to have some degree of metabolic protection [26], DBA/2 mice are 10% heavier and have decreased mean pancreatic islet area [27], 129 T2 mice have higher glucose levels following a glucose injection [27], and though both C57BL/6 and BFMI mic have reduced DNA methylation of the *Mc4r* gene, only BMFI mice have increased *Mc4r* expression [28]. Genetic background clearly affects the response to dietary fat, and thus it is important to determine if findings from the most commonly studied mouse strains are replicated in other strains before expecting the research to be informative for human health. Here, we investigated obesity epigenetics the Small (SM/J) strain of mouse, a strain that is less commonly studied overall but that our lab group has extensively characterized in the context of quantitative genetics, gene expression, and its obesogenic response to dietary fat [29–31].

DNA methylation is just one of several regulatory factors that control gene expression, thus we did not expect to find methylation changes in all differentially expressed genes. However, we were interested in genes where expression differences and methylation differences coincided, as these could make promising candidate genes for epigenetic therapies in the future. We tested the hypothesis that a high-fat diet would alter the expression and methylation of genes involved in obesity and diabetes. Additionally, we tested the hypothesis that there would be sex differences in the genes affected by a high-fat diet.

## Materials and methods

### Animal rearing

To investigate how a high-fat diet alters gene expression and methylation, we studied the inbred Small (SM/J) mouse strain from The Jackson Laboratory (Bar Harbor, Maine). The SM/J strain originated from a selective breeding experiment for small size at 60 days of age [32]. In response to the same high-fat diet as used in the present study we have previously shown that SM/J mice have reduced glucose tolerance [30], increased organ weights [30], and have 2137 differentially expressed transcripts in the liver as assessed by an Illumina® WG-6 v.2 BeadChip [31]. Fifteen males and 15 females born at Loyola University in Chicago were bred to produce 56 mice for the study population. The offspring were weaned onto a low-fat (LF) or high-fat (HF) diet at 3 weeks of age (16 HF females, 12 LF females, 18 HF males, and 10 LF males). The diets were designed to be as similar as possible in terms of nutrients and calories; however, 15% of the calories came from fat in the LF diet (Research Diets D12284), whereas 42% did in the HF diet (Harlan Teklad diet TD.88137) (Additional file 1: Table S1). Most of the fat in the LF diet came from polyunsaturated fat, whereas most of the fat in the HF diet came from saturated fat, which has been shown to increase expression of genes involved in inflammation and lipogenesis in mouse livers [33]. Previous work by Erich et al. [30] showed that SM/J mice consume the same amount of food whether they are on the HF diet or the LF diet. The mice were fed ad libitum and after weaning each mouse was housed with one other mouse of the same sex and diet in a cage containing a wooden gnawing block (Bio Serve), a red privacy hut (Alt Design), and a 2″ × 2″ cotton nestlet (Ancare). Procedures followed an approved Institutional Animal Care and Use Committee protocol (Project #1188, Loyola University).

### Obesity phenotypes

The mice were weighed weekly from 1 to 17 weeks of age. They underwent an intraperitoneal glucose tolerance test (IPGTT) at 15 weeks of age. All tests started with a 4-h fast at 6:00 am, followed by a tail snip to measure the baseline glucose level, and an intraperitoneal injection of glucose (1 mg/g body weight). Glucose measurements were then taken from tail blood at 30, 60, and 120 min after injection. At 16 weeks of age, the mice received an intraperitoneal insulin tolerance test (IPITT), with the same protocol as the IPGTT except that insulin was injected instead of glucose (0.75 mU insulin/g body weight). For both tests, the glucose values at the 4 different time points were used to calculate the area under the curve (AUC) using the trapezoidal summation method [34].

At 17 weeks of age, the mice were fasted for 4 h and sacrificed via carbon dioxide asphyxiation between 10:00 am and 2:00 pm. Blood from a cardiac puncture (0.5 mL) was centrifuged at 4 °C, and serum levels of insulin, leptin, triglycerides, glucose, cholesterol, and free fatty acids were measured. We performed the necropsies on ice and recorded the weights of the liver, heart, reproductive fat pad, kidneys, spleen, brown fat, and skeletal muscle (gastrocnemius). We weighed only the reproductive fat pad rather than all of the fat pads, because it is strongly genetically (h^2^ = 0.7–0.9) and phenotypically correlated (*r* = 0.67–0.82) with the other fat pads [9, 35]. We flash-froze liver tissue in liquid nitrogen for RNA extraction.

We performed multivariate analysis of variance (MANOVA) using the stats package in R to analyze differences in the obesity traits. Multivariate tests were performed on the following three groups of traits: weekly weights, diabetes-related traits (week 15 and 16 weights, baseline glucose during the IPGTT, IPGTT AUC, baseline glucose during the IPITT, and IPITT AUC), and necropsy traits (week 17 weight, organ weights, and serum biomarkers), as well as all the associated univariate tests (ANOVA). Differences were interpreted as significant for *p*-values less than 0.05.

### Gene expression

We extracted RNA from the liver tissue using the Qiagen RNeasy Plus Mini kit for RNA-seq with poly-A selection. A total of 21 libraries were sequenced, each with 2 mice of the same sex and diet pooled together. There were 6 LF female libraries, and 5 of each of the other sex-diet groups. A 1 × 50 single read sequencing run was done on an Illumina HiSeq 2500 machine (Illumina Inc.). The FastQ files were aligned to the Ensembl release 76 assembly using STAR version 2.0.4b [36]. The gene counts were then analyzed with the R package edgeR [37]; differences in library size were accounted for with a TMM normalization, and genes with counts of zero were filtered out. The weighted likelihoods were then calculated using the voom function in the R package Limma based on the mean-variance relationship of each gene and transcript. Generalized linear models were used to test for differential expression. We tested for the effect of sex, of diet, and of a sex*diet interaction on gene expression. Any gene with a false discovery rate (FDR) adjusted q-value of 0.05 or less was considered differentially expressed. We performed a pathway analysis using the R package GAGE [38] to identify pathways that were significantly up-regulated, down-regulated, or perturbed in both directions. We visualized the pathways with the R package Pathview [39].

We validated the differential expression for 3 genes in the females (*Adam11*, *Lad1*, and *Galnt10*) and 3 in the males (*Adam11*, *Abcg8*, and *Col1a1*) using rt-qPCR, with *Gapdh* as a normalizer (Additional file 1: Table S2). To do this, we extracted total RNA from the livers of 3 HF and 3 LF mice of each sex using Tri-Reagent (MRC), following the manufacturer’s instructions. The concentration and quality of the RNA from each sample was assessed twice with a NanoDrop Spectrophotometer, and only samples with a 260/280 ratio between 1.7–2.1 and a 260/230 ratio between 2.0–2.4 were used. We then immediately reverse transcribed the RNA to cDNA using the High-Capacity cDNA Reverse Transcription Kit (Applied Biosystems), following the manufacturer’s instructions. Primers were selected from the literature, and if none were found we used PrimerBank to design the primers. All primers were synthesized by Thermo Fisher Scientific (the sequences are listed in Additional file 1: Table S3). We performed RT-qPCR using 10 μL of PowerUp™ SYBR® Green Master Mix (Thermo Fisher), 1 μL of the forward primer, 1 μL of the reverse primer, 4 μL of 20-fold diluted cDNA, and 4 μL of water, with a total volume of 20 μL for each reaction. The RT-qPCR was performed with a StepOnePlus Real-Time PCR System (Applied Biosystems) at the following conditions: 20 s at 95 °C, followed by 40 cycles of 3 s at 95 °C and 30 s at 60 °C. For each of the 3 biological replicates, 3 technical replicates were used, along with a no-template control and a no-reverse-transcriptase control. We did a relative quantification of each gene using *Gapdh* as a reference using the comparative ΔΔCt method. There were no differences in *Gapdh* expression between the diet treatment groups. The expression data is available on NCBI’s Gene Expression Omnibus (GEO), record number GSE121525.

### DNA methylation

We performed a phenol-chloroform extraction to isolate DNA from the liver tissue. Genome-wide DNA methylation was then assessed with Methylated DNA Immunoprecipitation Sequencing (MeDIP-seq) and Methylation-sensitive Restriction Enzyme Sequencing (MRE-seq), as described by Li et al. [40]. MeDIP-seq detects methylated sites while MRE-seq detects unmethylated sites, and when used in combination these two techniques provide a single CpG resolution methylation map that has high concordance with whole-genome bisulfite sequencing at only a fraction of the cost [41]. The sequencing was done by running 2 × 75 bp paired-end reads via Illumina NextSeq 500. Four mice of the same sex and diet treatment were pooled per library, yielding 2 biological replicates per group. The NIH Epigenomics Project recommends using 2 replicates with a combined total coverage of 30x for whole genome bisulfite sequencing [46], although this can quickly become cost prohibitive for larger sample sizes. When MRE-seq and MeDIP-seq are combined they have comparable coverage to whole genome bisulfite sequencing [39], making 2 replicates sufficient here.

To synthesize the MRE-seq and MeDIP-seq data and test for differential methylation, we used the R package methylMnM, which was specifically designed for this purpose. First, we split the mouse mm9 genome into 500-base-pair windows (for a total of 5,283,825 windows); then, we assessed the proportion of methylated CpGs in each window; and from there we calculated the novel M&M test statistic to determine if the methylation level was different between the two diet treatments [42]. M&M tests two groups at a time, which yielded 4 pairwise comparisons of the female libraries and 4 of the males. To synthesize the information from all 4 library comparisons per sex, we used Fisher’s combined probability test [43]. To examine DMRs due to diet in the females, the *p*-value from the M&M test comparing the first HF-female library with the first LF-female library was combined with the p-value from the M&M test comparing the second HF-female library with the second LF-female library according to the following equation by Fisher:

$$ {X}_{2k}^2\sim -2\sum \limits_{i=1}^k\ln \left({p}_i\right) $$

In this case, *p*_*i*_ is the *p*-value from the pairwise M&M test, and k is the number of tests combined. We calculated a combined *p*-value for each 500-base-pair window, corrected for FDR with the Benjamini-Hochberg method [44], and calculated how many of these windows were differentially methylated based on q-value cutoffs of less than 0.05 and 0.01.

For each DMR, we then identified the nearest gene to it, if it fell within a gene, if it fell within a promoter, if it contained a known regulatory element listed in Ensembl (mouse genome assembly GRCm38.p5) [45], and if the gene closest to it was already known to be involved in obesity, diabetes mellitus, or cardiovascular diseases based on Phenopedia’s continuously updated list of genes uncovered by genetic association studies in humans (downloaded May 7, 2017). We also classified the DMR as being either in an intergenic region, exon, intron, or promoter. This was done using the full list of introns, exons, and genes downloaded from the NCBI37/mm9 assembly on the UCSC Genome Browser. If a DMR overlapped both an intron and an exon, it was classified as falling within an exon. It was classified as a promoter if it was within 2000 base pairs upstream of a transcription start site or 600 base pairs downstream of one. To determine if the DMRs were associated with gene expression, we randomized the DMRs across the genome and calculated how many fell within differentially expressed genes due to chance. To account for the general underrepresentation of DMRs in intergenic regions during the randomization, the percent of DMRs that were allowed to be randomized into intergenic regions was equal to the percent that actually exist in those regions. We performed a chi-square test to determine if the DMRs were found in regulatory regions at a greater rate than expected due to chance.

Prior to combining the *p*-values with Fisher’s combined probability test, we compared the replicates, which represents technical and biological noise. There was one significant DMR when comparing the two LF male libraries (q < 0.05) and 21 significant DMRs when comparing the HF male libraries, whereas each comparison of an LF with an HF male library had between 22 and 66 DMRs. There were 84 significant DMRs when comparing the two LF female libraries with each other and 22 significant DMRs when comparing the HF female libraries, whereas each comparison of an LF with an HF female library had between 9 and 190 DMRs. Very few DMRs were found across multiple comparisons. To understand the meaningful methylation differences, it is important to use replicates, as we did here. The methylation data is available on NCBI’s Gene Expression Omnibus (GEO), record number GSE122016.

## Results

### Obesity phenotype

**Fig. 1:**
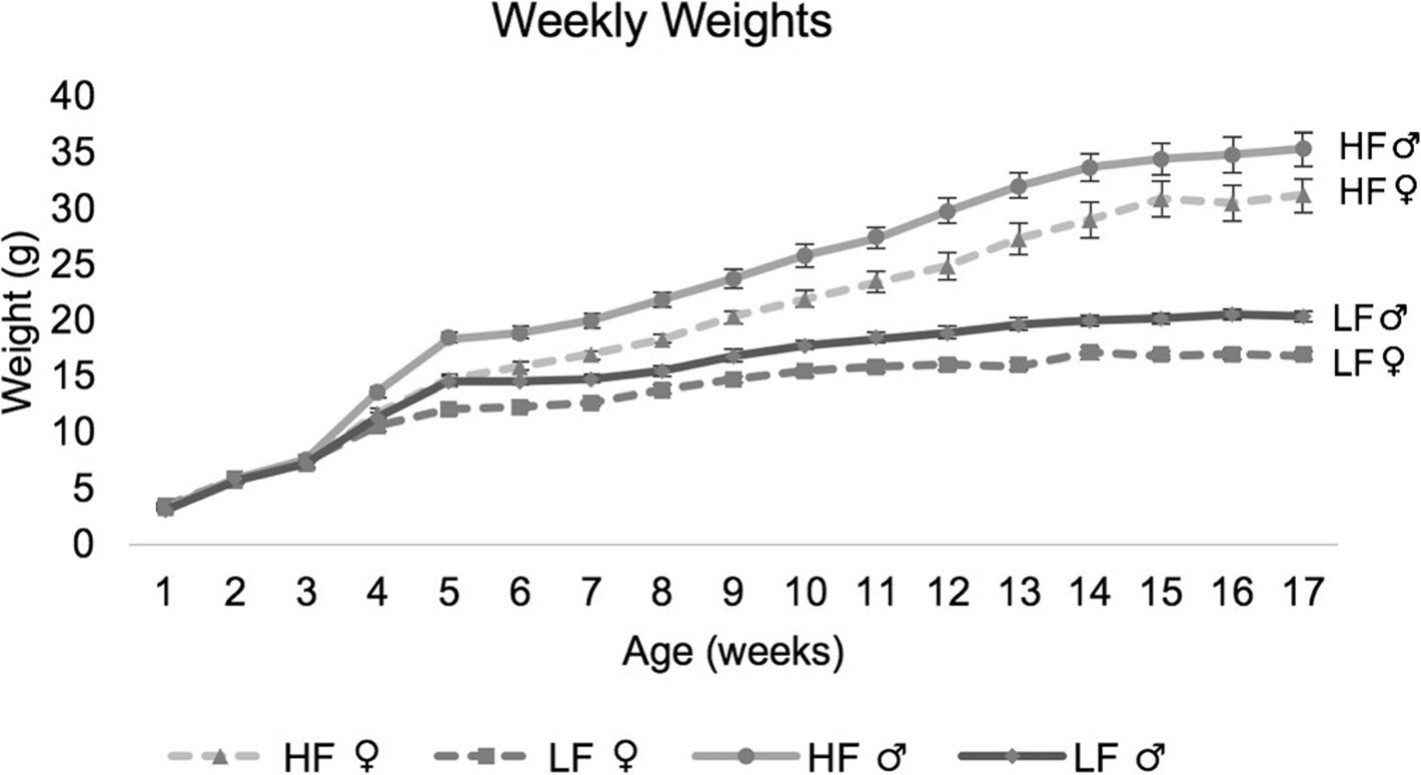
The average weight of mice in grams (± one standard error) from 1 to 17 weeks of age. Diet had a statistically significant effect from 4 weeks of age and on. HF = High-fat diet, and LF = Low-fat diet. LF male *n* = 10, HF male *n* = 18, LF female *n* = 12, HF female *n* = 16

**Fig. 2:**
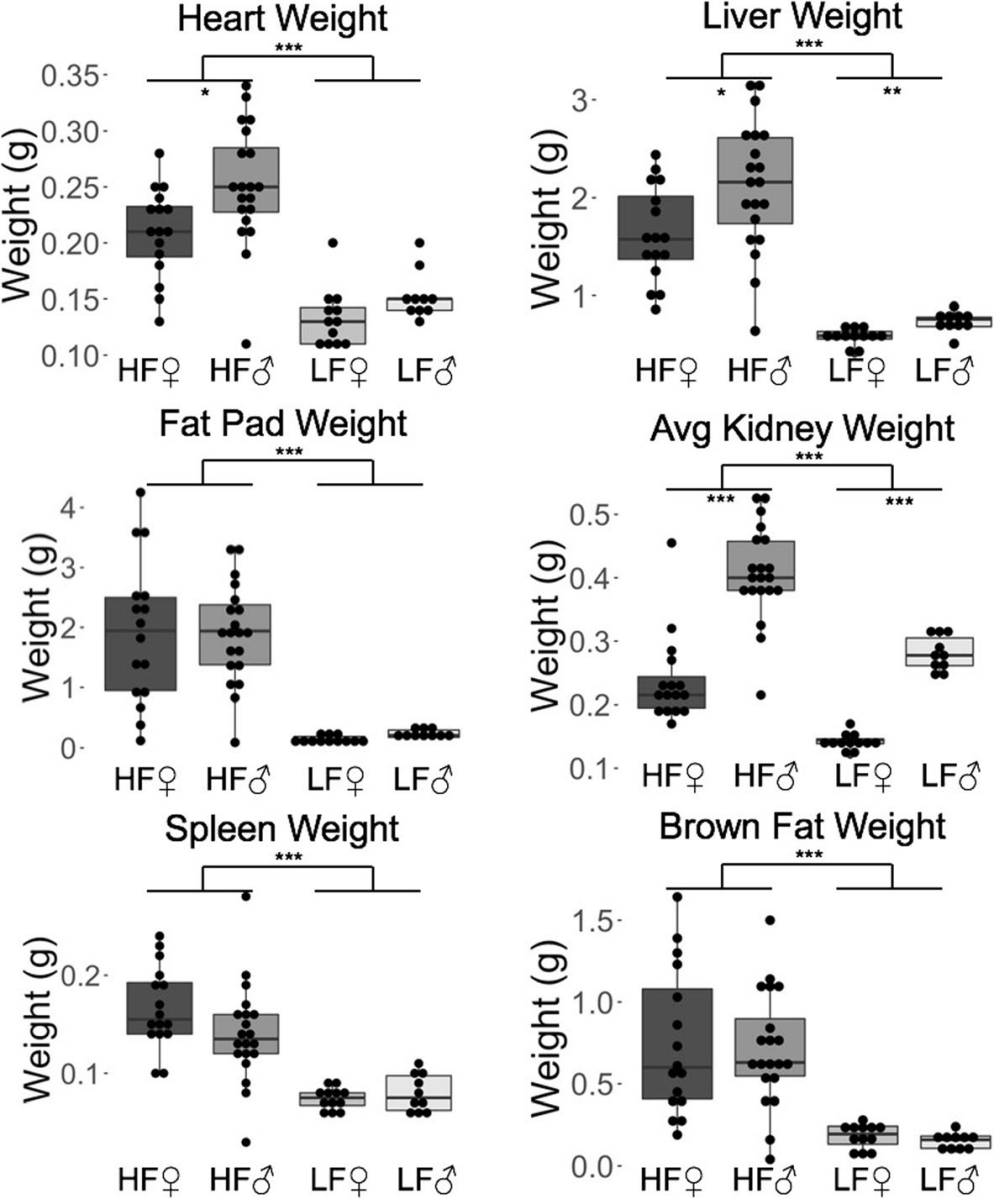
The average organ weights for each sex and diet group in grams. All organ weights were significantly heavier in the high-fat mice than the low-fat mice. The liver, heart, and kidneys were heavier in males than females. HF = High-fat diet, and LF = Low-fat diet. LF male n = 10, HF male n = 18, LF female n = 12, HF female *n* = 16. *** *p* < 0.001, ** *p* < 0.01, * *p* < 0.05, absence of asterisks indicates not-significant

Diet significantly affected the weekly weights (*p* = 1.59 × 10^− 7^), diabetes-related traits (*p* = 8.11 × 10^− 11^), and necropsy traits (*p* = 6.21 × 10^− 7^) (Additional file 1: Table S4). After only one week of being on the diet treatment (4 weeks of age), high-fat (HF) mice weighed significantly more than low-fat (LF) mice, and the difference became more pronounced with age (Fig. 1). The HF mice had reproductive fat pads that were more than 8 times larger than the LF mice. The ANOVA revealed an overall increase in all organ weights on an HF diet, including 2.8 times heavier livers (1.51 × 10^− 13^) and 1.6 times heavier hearts (2.39 × 10^− 8^) (Fig. 2, Additional file 1: Table S4).

**Fig. 3:**
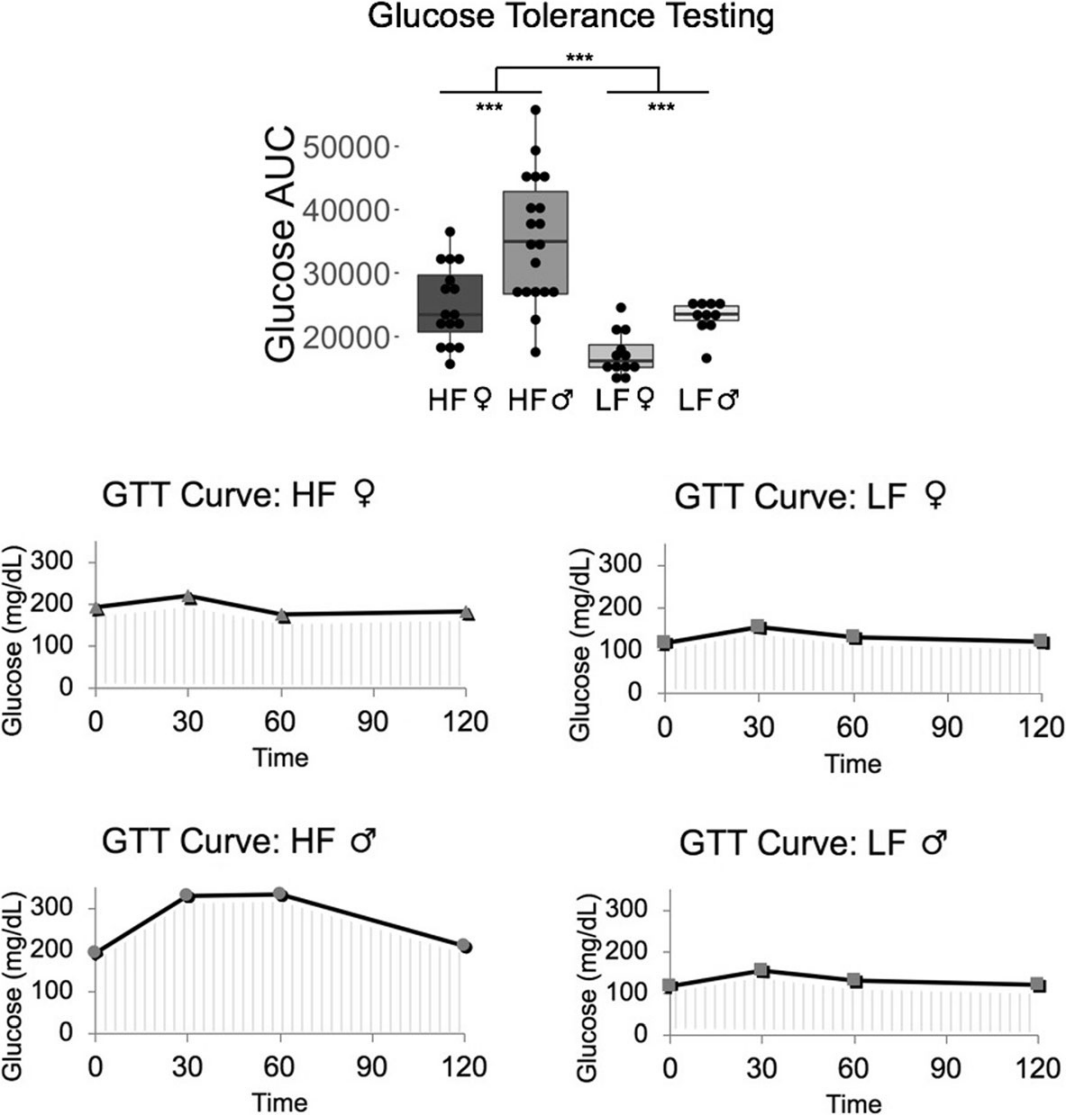
High-fat diet mice had an elevated response to glucose tolerance testing. HF = High-fat diet, and LF = Low-fat diet. LF male n = 10, HF male *n* = 18, LF female *n* = 12, HF female *n* = 16. *** *p* < 0.001, ** *p* < 0.01, * *p* < 0.05, absence of asterisks indicates not-significant

**Fig. 4:**
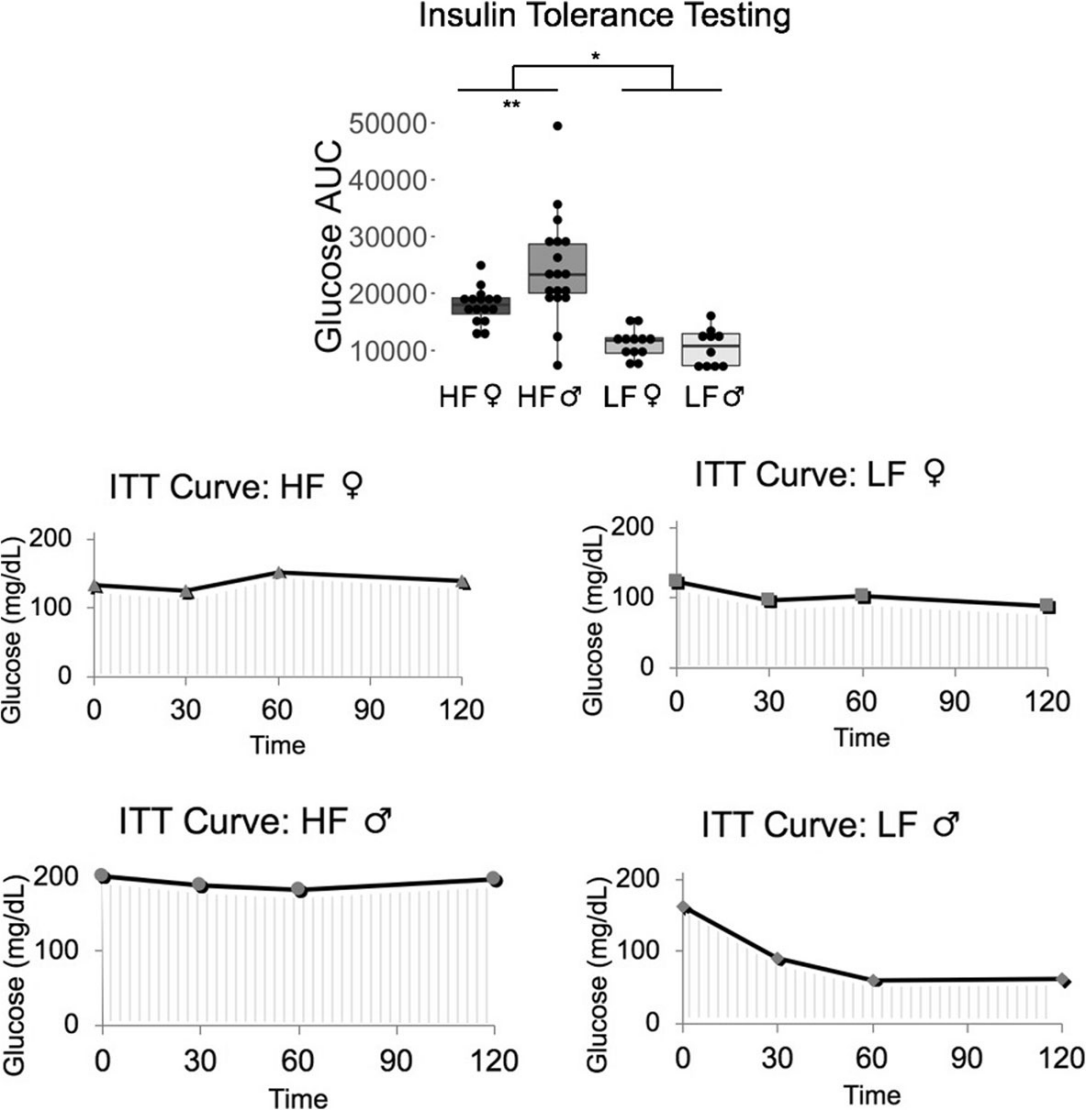
High-fat diet mice had a reduced sensitivity to insulin tolerance testing. HF = High-fat diet, and LF = Low-fat diet. LF male n = 10, HF male *n* = 18, LF female *n* = 12, HF female *n* = 16. *** *p* < 0.001, ** *p* < 0.01, * *p* < 0.05, absence of asterisks indicates not-significant

**Fig. 5:**
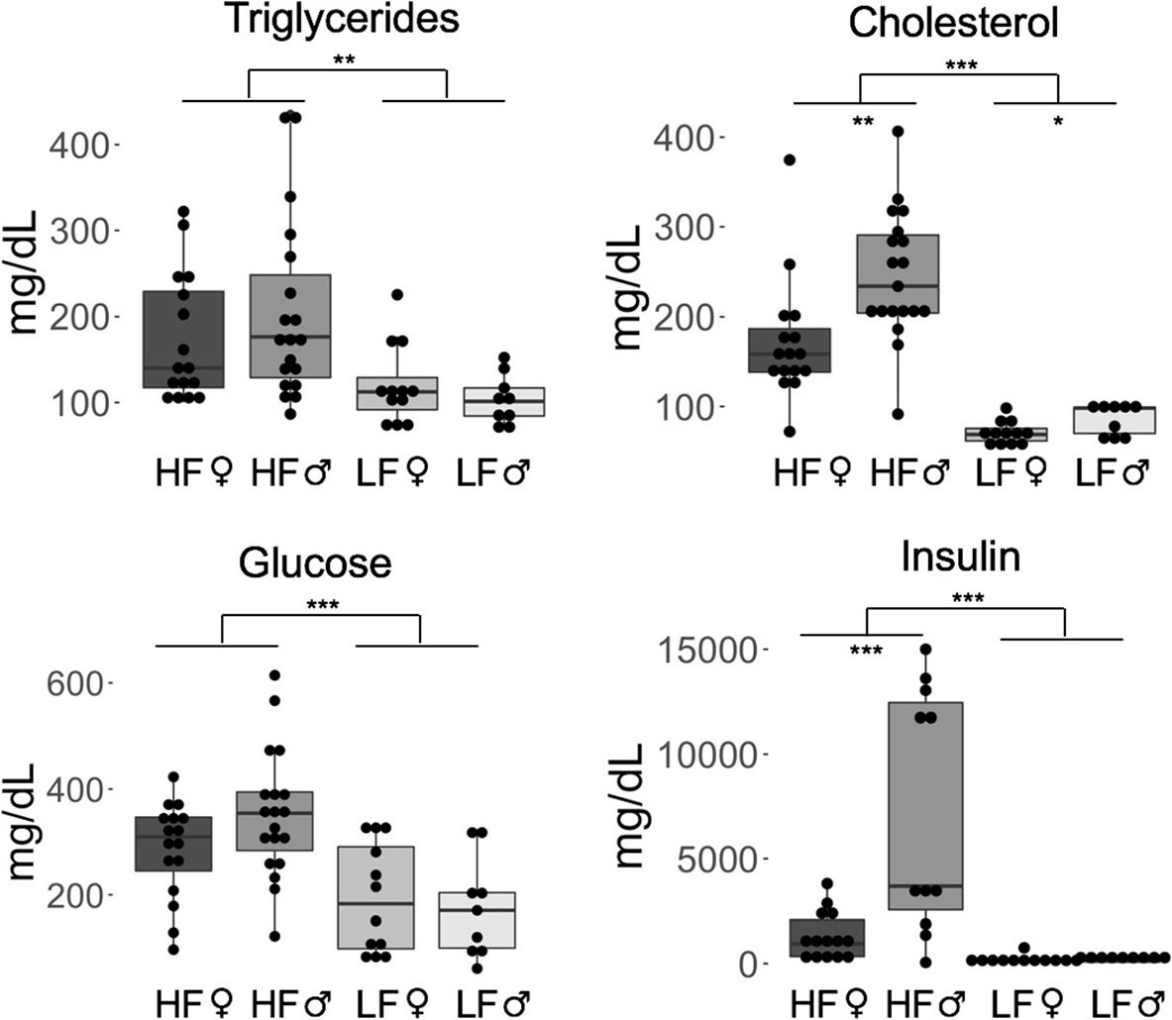
High-fat diet mice had higher levels of triglycerides, cholesterol, glucose, and insulin in their serum than low-fat diet mice. HF = High-fat diet, and LF = Low-fat diet. *** *p* < 0.001, ** *p* < 0.01, * *p* < 0.05, absence of asterisks indicates not-significant

Diet also significantly affected the response to intraperitoneal glucose and insulin tolerance testing, with HF mice having higher glucose area under the curve (AUC) values for both the IPGTT (*p* = 5.16 × 10^− 7^) (Fig. 3) and the IPITT (*p* = 2.53 × 10^− 8^) (Fig. 4), indicating impaired glucose and insulin signaling. Figures 3 and 4 depict curves of the mouse that had the median AUC value per sex and diet group to illustrate what the curves looked like. All serum biomarkers except for free fatty acids had higher levels due to an HF diet (Fig. 5), particularly in males.

**Fig. 6:**
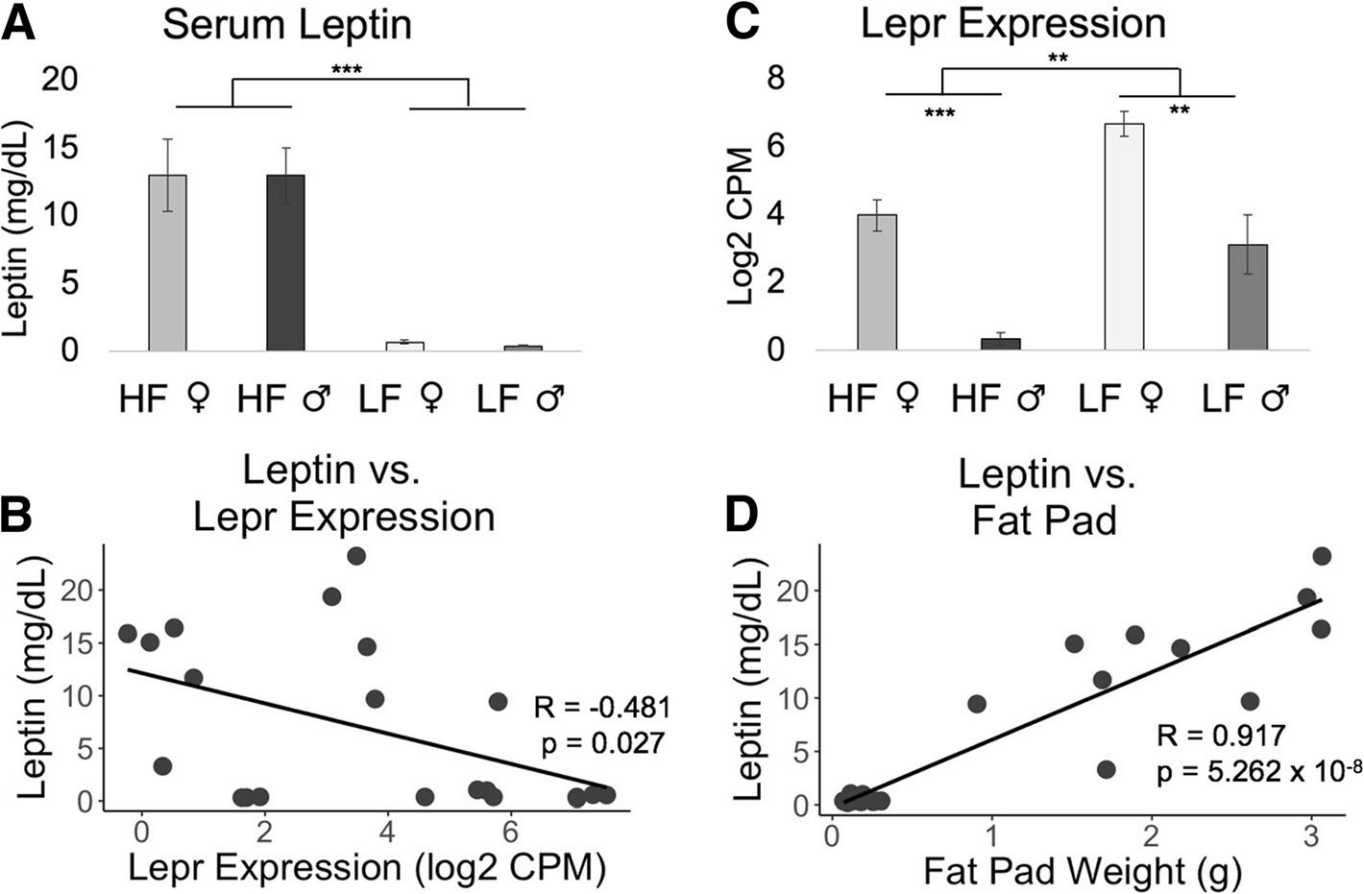
**a** A high-fat diet drastically increased leptin levels, **b** and was negatively correlated with hepatic expression of the leptin receptor *Lepr* gene. **c** High-fat-fed mice had signifcantly reduced expression of *Lepr*. **d** Serum leptin levels were positvely correlated with the weight of the reproductice fat pad. HF = High-fat diet, and LF = Low-fat diet. Error bars represent ± a single standard error. *** *p* < 0.001, ** *p* < 0.01, * *p* < 0.05, absence of asterisks indicates not-significant

HF mice had higher levels of cholesterol (*p* = 5.05 × 10^− 12^; 2.2 times higher in females, 3 times higher in males), triglycerides (*p* = 0.003; 1.3 times higher in females, 2.1 times higher in males), glucose (*p* = 1.35 × 10^− 4^; 1.4 times higher in females, 2 times higher in males), and insulin (*p* = 1.05 × 10^− 4^; 6.7 times higher in females, 38 times higher in males) (Fig. 5). HF mice also had substantially higher levels of leptin (*p* = 7.61 × 10^− 8^), with HF female mice having 20 times more leptin in their serum and HF male mice having 42 times more than LF mice (Fig. 6a). The correlation between serum leptin levels and the leptin receptor (*Lepr*) gene was negative (*R* = − 0.481, *p* = 0.027) (Fig. 6b), with HF mice having 7 times lower expression of *Lepr* in the liver than LF mice (Fig. 6c). Leptin had a strong positive correlation with fat pad weight (*R* = 0.917, *p* = 5.262 × 10^− 8^) (Fig. 6d).

**Fig. 7:**
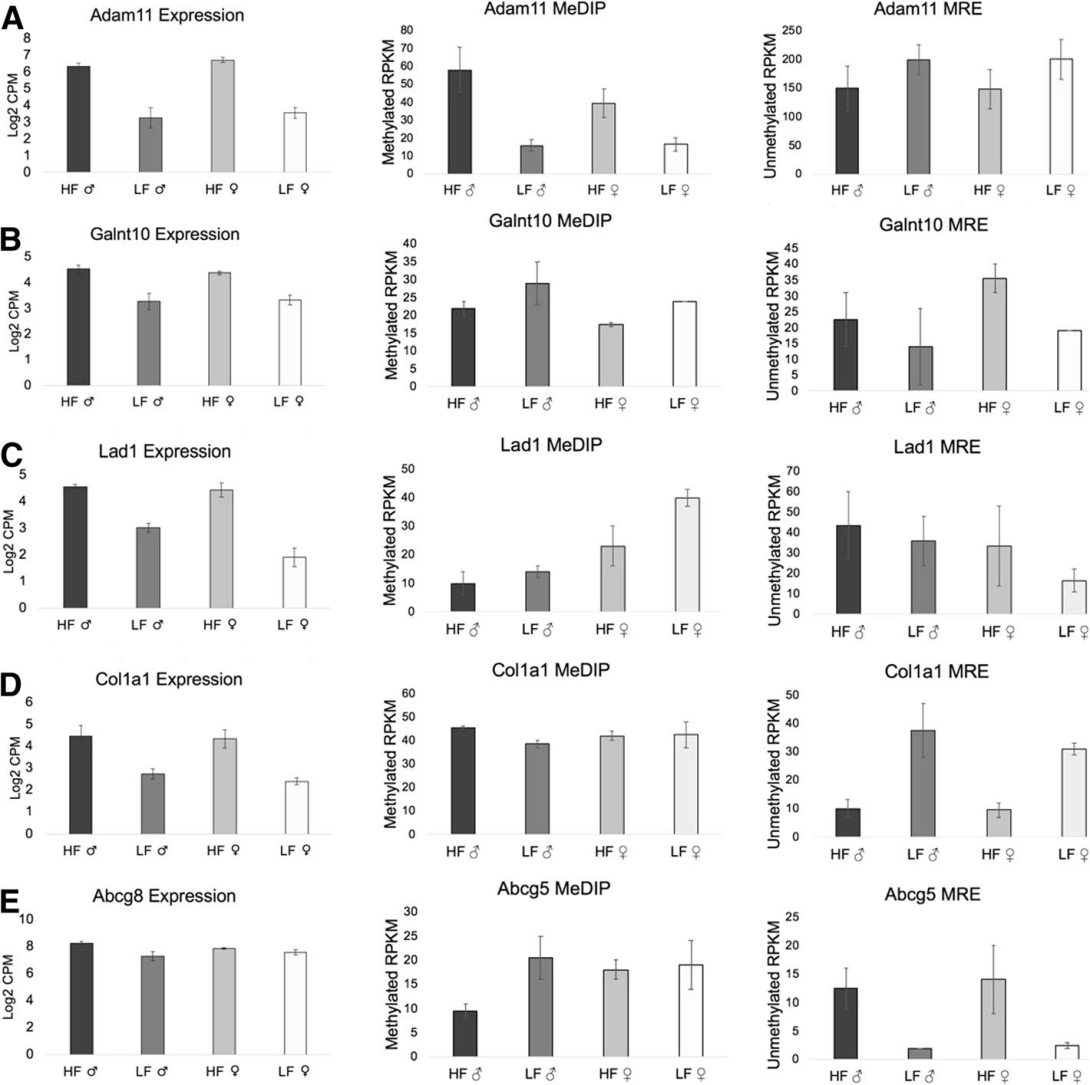
**a** High-fat (HF) diet mice had significantly higher methylation and higher *Adam11 *expression than low-fat (LF) diet mice. **b** HF mice also had higher *Galnt10* gene expression, with HF females having significantly less methylation in their first intron than LF females. **c** HF mice had higher expression of *Lad1*, and the first intron (also a promoter region) had lower methylation in HF females than LF females. **d** HF mice had higher expression of the *Col1a1 *gene than LF mice, with significantly increased methylation in HF males across exons 23 and 24 compared to LF males. **e **HF males had less methylation at a region between the genes *Abcg8* and *Abcg5*, which lie head-to-head, and the HF males had significantly increased expression of both *Abcg8* and *Abcg5*

The body weights (*p* = 4.17 × 10^− 5^), diabetes traits (*p* = 0.002), and serum biomarkers (*p* = 1.54 × 10^− 14^) were also significantly affected by sex. Irrespective of diet, males weighed more (*p* = 4.64 × 10^− 4^ at 17 weeks), had heavier livers (*p* = 0.014) and kidneys (*p* = 2.20 × 10^− 16^), and had higher glucose AUC values during glucose tolerance testing (*p* = 1.59 × 10^− 5^).

Although the sex-by-diet interaction effect was not significant on a multivariate level, it was significant on a univariate level for cholesterol (*p* = 0.005), insulin (*p* = 0.002), and glucose AUC during the intraperitoneal insulin tolerance test (IPITT) (*p* = 0.024).

### Gene expression

The multidimensional scaling (MDS) plot indicated that the gene expression libraries clustered primarily by sex (dimension 1, 74% of the variance) and then by diet (dimension 2) (Additional file 1: Figure S1). Diet altered the expression of 4356 genes in the liver (q < 0.05), or approximately one-fifth of the genome. The log2 fold changes of the significantly differentially expressed genes ranged from − 0.19 to − 5.83 and 0.20 to 7.17. More differentially expressed genes were detected in males (3330) than in females (1750). Of the genes that were differentially expressed, 848 were differentially expressed due to diet only in females and 2428 were unique to males (Additional file 1: Tables S5 and S6). There were 184 genes with significantly different expression due to a sex-by-diet interaction, which a GO Enrichment analysis (Gene Ontology Consortium) showed were enriched for three biological processes: epoxygenase P450 pathway (*p* = 2.36 × 10^− 5^), oxidation-reduction process (*p* = 5.58 × 10^− 5^), and response to stilbenoid (*p* = 5.21 × 10^− 3^).

The GAGE pathway analysis revealed that an HF diet changed the regulation of 7 pathways (Additional file 1: Table S7). This included the downregulation of the oxidative phosphorylation pathway and upregulation of the cytokine-cytokine pathway, indicating that the HF diet reduced mitochondrial function and increased inflammation (Additional file 1: Figure S2). In females, there were 4 pathways upregulated by an HF diet: cytokine-cytokine receptor interaction, chemokine signaling, cell adhesion molecules, and the natural killer cell mediated cytotoxicity pathways. In males, the cytokine-cytokine receptor interaction pathway was also upregulated by an HF diet, while the ribosome and oxidative phosphorylation pathways were downregulated (Additional file 1: Table S8). In females, 29 GO Biological Processes were upregulated, nearly all of them related to the immune system. Even more were upregulated in males, with 61 affected processes, again mostly involved in the immune system (Additional file 1: Table S9). No GO processes were downregulated, which was perhaps related to the strong upregulation of inflammation.

### Methylation

A q-value cutoff of 0.05 revealed tens of thousands of differentially methylated regions (DMRs) associated with diet, which encompassed 0.6–0.8% of the nearly 5.3 million 500-base-pair windows in the genome. A cutoff of 0.01 was more discriminating, with less than 0.04% of windows falling below it, allowing us to focus on a few thousand genes with differential methylation (Additional file 1: Tables S10 and S11). The comparison of HF and LF females resulted in 2356 DMRs (q < 0.01), which was more than the 1539 DMRs between the HF and LF males (Additional file 1: Table S12). There were even more DMRs due to sex than diet, with HF males and females differing at 3831 regions and LF males and females differing at 5632 regions (q < 0.01). A greater percentage of DMRs were found on the X chromosome in the between-sex comparisons (2.3–2.8%) than in the within-sex comparisons (0.1–0.3%, q < 0.01).

In all, 7814 genes (38.3% of genes) in the liver contained at least one diet-induced DMR (q < 0.05) between its outermost transcription start and end sites in the females, as did 7086 genes (34.7%) in the males (Additional file 1: Table S13). When the DMRs were assigned to one of four categories, 15% fell within promoters, 25% in exons, 34% in introns, and 31% were in intergenic regions. Not all of these categories were mutually exclusive, since several DMRs encompassed both exons and promoters. Many of the DMRs were in regulatory regions, including 10–12% in enhancers, 14–16% in promoters, 3–7% in CTCF transcription factor binding sites, and 34% in promoter flanking regions as defined by Ensembl (Table 1). Across the genome, only 4% of the windows overlapped enhancers, 2% overlapped CTCF binding sites, and 8% overlapped promoter flanking regions. DMRs were far more likely to be found in these regulatory regions than in non-regulatory regions of the genome (χ^2^, *p* < 1 × 10^− 10^).

**Table 1:** The distribution of significant differentially methylated regions (DMRs) (q < 0.01) across the genome associated with diet. Numbers indicate how many 500 base pair windows overlap each genomic region, with the percent of the total significant DMRs that overlap such regions in parentheses for the female and male mice. The percent of windows across the entire genome that overlap these genomic regions is listed as a comparison, illustrating the overrepresentation of regulatory regions in the DMRs

Region	Female DMRs	Male DMRs	Whole Genome
Enhancer	237 (10.0%)	180 (11.7%)	3.5%
CTCF Binding Site	157 (6.7%)	55 (3.6%)	1.7%
TF binding site	33 (1.4%)	12 (0.8%)	0.3%
Promoter Flanking Region	795 (33.8%)	522 (33.9%)	8.1%
Promoter	370 (15.7%)	215 (14.0%)	4.5%
Exon	598 (25.4%)	415 (27.0%)	7.5%
Intergenic	748 (31.8%)	471 (30.6%)	58.6%

Although only a small percentage of the DMRs fell in differentially expressed genes, it nevertheless happened more often than expected by chance (χ^2^, *p* = 2.2 × 10^− 8^). In the females, 2170 (5.6% of) DMRs fell within differentially expressed genes, whereas only 1994 (5.1%) were expected to by chance. In the males, 3209 (10.2% of) DMRs fell within differentially expressed genes, whereas only 2992 (9.5%) were expected to by chance.

### Candidate genes with differential methylation and differential expression

The differentially expressed gene with the lowest q-value in the females was ADAM metallopeptidase domain 11 (*Adam11*) (q = 7.6 × 10^− 20^), which also had three adjacent DMRs encompassing exons 14–18 of the gene. The males had the same DMRs and exhibited the same pattern as the females of having higher methylation and higher *Adam11* expression due to an HF diet (Fig. 7a). Although the *Adam11* gene is known to be expressed in the mouse liver (MGI Gene Expression Database), its role in obesity and diabetes has not been discussed. It belongs to a family of genes involved in cell signaling, migration, and adhesion, and mice lacking *Adam11* have impaired spatial learning and motor coordination, along with a reduced response to inflammatory pain [47]. Perhaps the increased inflammation associated with obesity leads to an increase in inflammation-related pain.

An HF diet also increased expression of the UDP-N-acetyl-alpha-D-galactosamine:polypeptide N-acetylgalactosaminyltransferase 10 (*Galnt10*) gene in both the males and the females. A DMR in its first intron was significantly less methylated by an HF diet in the females (the same trend was observed in the males, but was not significant) (Fig. 7b). Genome-wide association studies have found SNPs in *Galnt10* that are associated with BMI [48] and physical activity [49]. GALNT10 catalyzes the synthesis of mucin-type O-glycosylation, a type of post-translational modification. Important mucin-type O-linked glycoproteins include interluekin-2 and proteins involved in homing leukocytes to inflamed areas [50].

An HF diet was also associated with increased expression of the ladinin 1 (*Lad1*) gene in the males and females, accompanied by decreased methylation in the females (with a non-significant trend in the males) at two adjacent DMRs in its first intron, which is also a promoter region (Fig. 7c). *Lad1* is a part of the basement membrane, which increases around liver vessels in liver fibrosis. Basement membrane peptide levels increase in the serum as the severity of liver damage increases [51]. This is relevant because the HF mice had visibly fattier livers, in line with the increased risk of non-alcoholic fatty liver disease (NAFLD) due to obesity.

Further epigenetic evidence of liver distress induced by an HF diet is the upregulation of the collagen type I alpha 1 chain (*Col1a1*) gene in HF males and females, accompanied by increased methylation in the males at a DMR spanning exons 23 and 24 of the gene (Fig. 7d). COL1A1 is a subunit of type 1 collagen, which accumulates in the liver during fibrosis and cirrhosis. When Calvente et al. [52] administered siRNA to degrade transcripts of *Col1a1* in mice with advanced liver fibrosis, collagen deposition decreased by half and several other profibrogenic genes were downregulated. Our results support the notion that siRNA or other epigenetic treatments for elevated *Col1a1* levels may help in obesity-related liver fibrosis.

The ATP binding cassette subfamily G member 5 (*Abcg5*) gene lies head-to-head with *Abcg8*, and both were expressed higher in HF males than LF males. A DMR close to the start of both genes, located in the first intron of *Abcg5*, had lower methylation due to an HF diet and may be involved in the co-regulation of the genes (Fig. 7e). They encode proteins forming a heterodimer that facilitates the excretion of cholesterol into bile. Mutations in either gene are associated with atherosclerosis and sitosterolemia, a condition that leads to cardiovascular disease through the accumulation of sterols [53]. Our findings support previous studies that have identified the upregulation of *Abcg5* and *Abcg8* in response to insulin resistance and an HF diet [53–56]. The upregulation of the heterodimer may show an attempt to eliminate the excess cholesterol from the body, although HF mice still had 2–3 times as much serum cholesterol as LF mice.

## Discussion

The results supported our hypothesis that a high-fat (HF) diet would alter the expression and methylation of genes involved in obesity and diabetes. The results also supported our hypothesis that the set of genes affected by an HF diet in males and females would not completely overlap. In the females, 2170 (5.6%) of differentially methylated regions (DMRs) (q < 0.05) occurred within genes that were differentially expressed due to diet, whereas in males 3209 (10.2%) of DMRs did.

An HF diet was associated with drastic alterations in DNA methylation, gene expression, and physiology in the SM/J mice. By 17 weeks of age, mice on an HF diet weighed 70% more than mice on a low-fat (LF) diet, which we interpret as obesity. An HF diet significantly: increased all body weights and organ weights; decreased glucose and insulin tolerance; and increased serum levels of cholesterol, triglycerides, glucose, leptin, and insulin. The HF diet did not increase levels of free fatty acids in the serum, a trend that Do et al. [56] also found in C57BL/6 J mice, despite elevated fatty acid levels in the liver.

The expression of 4356 genes and the methylation of more than 7000 genes in the mouse liver were associated with diet. More than one-third of genes had at least one DMR associated with diet. The DMRs occurred in regulatory regions such as enhancers, transcription factor binding sites, and promoter flanking regions significantly more often than these regions occur in the genome, supporting the notion that methylation plays an important role in regulating the response to an HF diet. That role is not straightforward, however. The DMRs fell within differentially expressed genes significantly more often than expected by chance; however 31% of the DMRs did not even occur within genes at all, although 17% of those overlapped with enhancers or transcription factor binding sites. This is on par with the findings of Rönn et al. [21], who noted that fewer than 3% of adipose tissue DMRs following 6 months of exercise were located in differentially expressed genes in men. Methylation is only one piece of the puzzle; it will be interesting in the future to correlate the DNA methylation changes with histone modifications, as these two regulatory features work together to modulate gene expression.

Our gene expression findings mostly support those of other mouse studies, while highlighting differences that can be caused by factors such as genetic background, percent of fat in diet, and type of fat in the diet. For instance, Do et al. [56] compared liver expression of HF and LF male C57BL/6 J mice and found that an HF diet perturbed genes that were enriched for processes involved in immune and inflammatory response. Of the 332 genes they found differentially expressed due to an HF diet, 120 were the same as the ones we identified in the males (they did not study females). These included *Adam11*, *Abcg5*, and *Abcg8*, which we highlighted here as having differences in both expression and methylation associated with diet. We found 28 genes in common with Kim et al. [57], who identified 97 differentially expressed genes due to an HF diet in C57BL/6 J males. We found more genes in common with Kim et al. [57] and Do et al. [56] than either did with each other, even though they used the same mouse strain, which shows the utility of RNA-seq data over microarray data when comparing across studies. Of the 309 differentially expressed genes that Kirpich et al. [58] identified in male C57BL/6 mice due to an HF diet, we found 124 of the same genes. Kirpich et al. [58] shared 12 genes in common with Kim et al. [57] and 57 genes in common with Do et al. [56], and the only genes found in all three studies were *Nsdhl* and *Sqle* (Additional file 1: Figure S3). This highlights the difficulty of repeatability in gene expression studies. Like Inoue et al. [59] found in C57BL/6Ncrj male mice, we found that *Pparg* and its target gene *Cd36* were both upregulated in the male and female HF mice, corroborating their conclusion that an HF diet induces liver steatosis by upregulating *Pparg*. Similar to other studies, we found an upregulation of genes in pathways associated with defense, stress, and inflammation responses [56, 57].

We compared our list of differentially expressed genes with those found in 9 other mouse strains exposed to an HF diet by Shockley et al. [60] and found 16–27 genes in common with the males in each study and 3–15 genes in common with the females (Additional file 1: Table S14). The strain dependent results underscore the importance of studying obesity in multiple strains of mice instead of basing conclusions off of one strain. Differences in duration of diet treatment [61], type of fat [62], and percent of fat in the diets [63] also can affect gene expression and may contribute to variation across studies.

Replicating DMRs across studies can be even more difficult than replicating gene expression, since methylation can be more variable and fewer studies have investigated it, especially genome-wide. Ge et al. [64] found that *Lep* had lower hepatic expression and higher promoter methylation in HF-fed female CD-1 mice. Here, we also found a hypermethylated DMR in the *Lep* promoter of the females (q = 0.02), but no difference in expression. Ge et al. [46] additionally found a hypomethylated *Pparα* promoter, and although we too found a DMR in *Pparα*, ours was hypermethylated in HF mice, it was located in the second intron, and the gene was not differentially expressed. Yoon et al. [65] identified hypomethylated CpG sites 1.5-kb upstream of the *Casp1* gene in C57BL/6 N male mice, but we found no DMRs there or in that gene. Like them, we did find lower expression of *Ndufb9* in HF males along with a DMR, but our DMR was hypomethylated by an HF diet whereas theirs was hypermethylated. As exemplified by this variability across studies, understanding the methylation changes underlying obesity will require much more research in the context of multiple genetic backgrounds.

Males and females responded differently to an HF diet. Compared to HF females, HF males had higher cholesterol, higher insulin, and higher glucose AUC during the intraperitoneal insulin tolerance test. The sex differences were visible on the levels of methylation and gene expression as well. There were more differentially expressed genes associated with diet in the males (3330) than in the females (1750), and more than 2000 genes were differentially expressed only in the males. The 184 genes with a significant sex-by-diet interaction were enriched for the epoxygenase P450 pathway, oxidation-reduction process, and response to stilbenoid, suggesting sex differences in these pathways mediate the difference between the male and female response to dietary fat. Cytochrome P450 genes are important for homeostasis and encode enzymes involved in metabolizing fatty acids and drugs, so sex differences in this pathway are relevant to pharmaceutical approaches to weight loss. Likewise, a sex-by-diet effect on the response to stilbenoids is interesting because they have been shown to regulate lipids, and Lin et al. [66] found that the stilbenoid TSG prevented NAFLD in HF-fed rats, with results that hinted at a small but inconclusive difference between males and females. Although there were more differentially expressed genes due to diet in males than in females, the opposite was true for DMRs. However, while there were fewer DMRs total in males, more of their DMRs occurred within genes that were differentially expressed due to diet.

## Conclusions

This study identified thousands of genes that were differentially expressed and differentially methylated in response to a high-fat diet in SM/J mice. Genome-wide studies such as this are essential for developing a better understanding of the relevant epigenetic changes in obesity and identifying new targets for treatments. It is crucial that these treatments take sex into consideration, since—from the level of methylation to expression to obesity traits—males and females responded quite differently to an obesogenic diet.

## Additional file

Additional file 1: Supplement. (ZIP 4328 kb)
