# Supplementary material for: A high-fat diet alters genome-wide DNA methylation and gene expression in SM/J mice
Source: BMC Genomics. 2018 Dec 7;19:888. doi: 10.1186/s12864-018-5327-0 (PMC6286549; doi:10.1186/s12864-018-5327-0)
Supplement: Supplementary file 1 — Supplement. (ZIP 4328 kb) [file 12864_2018_5327_MOESM1_ESM.zip › Supporting Information.docx]

Table S1. Diet compositions.

| **Component** | **High-Fat Diet** | **Low-Fat Diet** |
| --- | --- | --- |
| Energy from fat, % | 42 | 15 |
| Casein, g/kg | 195 | 197 |
| Sugars, g/kg | 341 | 307 |
| Corn starch, g/kg | 150 | 313 |
| Cellulose, g/kg | 50 | 30 |
| Corn oil, g/kg | 0 | 58 |
| Hydrogenated coconut oil, g/kg | 0 | 7 |
| Anhydrous milk fat, g/kg | 210 | 0 |
| Cholesterol, g/kg | 1.5 | 0 |
| Kilojoules per gram | 18.95 | 16.99 |

Table S2. Primers used for RT-qPCR.

| **Gene** | **Forward primer** | **Reverse primer** |
| --- | --- | --- |
| *Adam11* | 5'-TGCTGCTGTTACCGCTTCT-3' | 5'-TCAGAGCCCTCTGGACTCTCT-3' |
| *Lad1* | 5'-ATGTCGGTCAGCAGAAAGGAC-3' | 5'-CTGTGGTTGAACTCAGGTTGC-3' |
| *Galnt10* | 5'-TGACCGATGCCGAGAGAGT-3' | 5'-AGAGAGCGATTCAGGGAGATT-3' |
| *Abcg8* | 5'-GTACGTGGGGTGTCCGGGGGTGAG-3' | 5'-GCGAGGCTGGTGGAGGGAGATGAG-3' |
| *Col1a1* | 5'-GCTCCTCTTAGGGGCCACT-3' | 5'-CCACGTCTCACCATTGGGG-3' |
| *Gapdh* | 5'-ACAATGAATACGGCTACAGCAACAG-3’ | 5’-GGTGGTCCAGGGTTTCTTACTCC-3’ |

Table S3. The RT-qPCR validation results. Values are presented as high-fat diet expression relative to low-fat diet expression levels. Fold differences were calculated with the ∆∆CT method and reported as a range to include the standard deviation. HF= High-fat diet, LF= Low-fat diet.

|  | Gene | Fold difference of HF vs. LF |
| --- | --- | --- |
| Females | *Adam11* | 6.57 ± 0.33 |
|  | *Lad1* | 3.54 ± 0.37 |
|  | *Galnt10* | 4.15 ± 1.80 |
| Males | *Adam11* | 7.84 ± 4.10 |
|  | *Col1a1* | 9.90 ± 6.08 |
|  | *Abcg8* | 11.77 ± 7.30 |

Table S4. The effect of diet and sex on weekly weights, diabetes traits, and organ weights. Averages are listed ± the standard error. LF male n = 10, HF male n = 18, LF female n = 12, HF female n = 16. P-values less than 0.05 are bolded.

| Group of Traits | Trait | Diet  p-value | Sex  p-value | Sex*Diet  p-value | LF ♀  Average | HF ♀  Average | LF ♂  Average | HF ♂  Average |
| --- | --- | --- | --- | --- | --- | --- | --- | --- |
| Weekly Weights | Weekly Weight MANOVA | **1.59E-07** | **4.17E-05** | 0.6673 |  |  |  |  |
|  | Week 1 (g) | 0.899 | 0.624 | 0.555 | 3.5 ± 0.3 | 3.4 ± 0.2 | 3.3 ± 0.2 | 3.3 ± 0.2 |
|  | Week 2 (g) | 0.905 | 0.679 | 0.670 | 6.0 ± 0.3 | 5.9 ± 0.3 | 5.9 ± 0.2 | 6.1 ± 0.3 |
|  | Week 3 (g) | 0.867 | 0.535 | 0.447 | 7.5 ± 0.5 | 7.3 ± 0.4 | 7.4 ± 0.3 | 7.7 ± 0.4 |
|  | Week 4 (g) | **0.002** | **0.004** | 0.298 | 10.7 ± 0.5 | 11.9 ± 0.5 | 11.4 ± 0.5 | 13.7 ± 0.5 |
|  | Week 5 (g) | **4.86E-08** | **3.21E-09** | 0.284 | 12.2 ± 0.4 | 14.8 ± 0.4 | 14.7 ± 0.6 | 18.5 ± 0.5 |
|  | Week 6 (g) | **1.72E-10** | **2.49E-07** | 0.537 | 12.5 ± 0.3 | 16.0 ± 0.4 | 14.6 ± 0.3 | 19.0 ± 0.6 |
|  | Week 7 (g) | **2.68E-12** | **3.17E-07** | 0.484 | 12.8 ± 0.4 | 17.0 ± 0.4 | 14.9 ± 0.4 | 20.1 ± 0.6 |
|  | Week 8 (g) | **6.30E-13** | **7.01E-07** | 0.180 | 13.9 ± 0.4 | 18.5 ± 0.4 | 15.7 ± 0.5 | 22.0 ± 0.7 |
|  | Week 9 (g) | **1.79E-12** | **1.05E-05** | 0.494 | 14.9 ± 0.3 | 20.4 ± 0.6 | 17.0 ± 0.5 | 23.8 ± 0.8 |
|  | Week 10 (g) | **1.59E-12** | **1.74E-05** | 0.497 | 15.6 ± 0.4 | 22.0 ± 0.7 | 17.9 ± 0.4 | 25.9 ± 1.0 |
|  | Week 11 (g) | **9.11E-01** | **5.84E-05** | 0.446 | 15.9 ± 0.4 | 23.6 ± 1.0 | 18.5 ± 0.5 | 27.5 ± 1.0 |
|  | Week 12 (g) | **1.77E-12** | **2.55E-05** | 0.358 | 16.1 ± 0.4 | 25.1 ± 1.2 | 19.1 ± 0.5 | 29.9 ± 1.1 |
|  | Week 13 (g) | **1.69E-13** | **4.70E-05** | 0.651 | 16.1 ± 0.4 | 27.5 ± 1.4 | 19.8 ± 0.5 | 32.2 ± 1.2 |
|  | Week 14 (g) | **2.13E-13** | **2.70E-04** | 0.501 | 17.2 ± 0.5 | 29.1 ± 1.5 | 20.2 ± 0.5 | 33.8 ± 1.3 |
|  | Week 15 (g) | **1.26E-14** | **0.001** | 0.848 | 17.0 ± 0.4 | 31.1 ± 1.6 | 20.3 ± 0.5 | 34.5 ± 1.4 |
|  | Week 16 (g) | **2.07E-13** | **0.001** | 0.786 | 17.1 ± 0.5 | 30.6 ± 1.6 | 20.6 ± 0.4 | 35.0 ± 1.6 |
|  | Week 17 (g) | **4.38E-14** | **4.64E-04** | 0.702 | 17.0 ± 0.4 | 31.3 ± 1.5 | 20.5 ± 0.5 | 35.5 ± 1.5 |
| Diabetes-related traits | Diabetes traits MANOVA | **8.11E-11** | **0.001658** | 0.13292 |  |  |  |  |
|  | Week 15 weight (g) | **5.85E-15** | **0.009** | 0.848 | 17.0 ± 0.4 | 31.1 ± 1.6 | 20.3 ± 0.5 | 34.5 ± 1.4 |
|  | Baseline glucose (mg/dL) | **5.55E-07** | **0.018** | 0.654 | 111.1 ± 4.5 | 165.1 ± 4.9 | 125.4 ± 4.8 | 183.7 ± 9.1 |
|  | GTT AUC | **5.16E-07** | **1.59E-05** | 0.194 | 16683 ± 598 | 23521 ± 942 | 20138 ± 1001 | 33880 ± 2121 |
|  | Week 16 weight (g) | **9.02E-14** | **0.005** | 0.786 | 17.1 ± 0.5 | 30.6 ± 1.6 | 20.6 ± 0.4 | 35.0 ± 1.6 |
|  | Baseline glucose (mg/dL) | **3.43E-06** | **0.016** | 0.102 | 116.3 ± 4.1 | 157.2 ± 5.2 | 134.6 ± 4.4 | 197.8 ± 13.1 |
|  | ITT AUC | **2.53E-08** | **0.019** | **0.024** | 9683 ± 645 | 9684 ± 645 | 9685 ± 645 | 9686 ± 645 |
| Necropsy traits | Necropsy Traits MANOVA | **6.21E-07** | **1.54E-14** | 0.1398 |  |  |  |  |
|  | Week 17 Weight (g) | **1.09E-11** | **0.032** | 0.892 | 17.0 ± 0.4 | 31.3 ± 1.5 | 20.5 ± 0.5 | 35.5 ± 1.5 |
|  | Leptin (mg/dL) | **7.61E-08** | 0.945 | 0.943 | 0.6 ± 0.1 | 12.9 ± 2.7 | 0.4 ± 0.1 | 12.9 ± 2.0 |
|  | Insulin (mg/dL) | **1.05E-04** | **0.001** | **0.002** | 189.9 ± 57.1 | 1274.4 ± 307.3 | 221.2 ± 55.0 | 7162.3 ± 1739.0 |
|  | Triglycerides (mg/dL) | **0.003** | 0.885 | 0.247 | 121.2 ± 13.4 | 173.6 ± 18.6 | 103.5 ± 9.6 | 204.0 ± 23.9 |
|  | Cholesterol (mg/dL) | **5.05E-12** | **5.63E-05** | **0.005** | 70.9 ± 3.6 | 171.9 ± 17.0 | 85.7 ± 5.6 | 245.9 ± 16.4 |
|  | Glucose (mg/dL) | **1.35E-04** | 0.138 | 0.063 | 193.9 ± 29.4 | 286.0 ± 22.9 | 175.9 ± 31.5 | 352.6 ± 27.6 |
|  | Free Fatty Acids (mg/dL) | 0.404 | 0.440 | 0.322 | 1.5 ± 0.1 | 1.3 ± 0.1 | 1.3 ± 0.1 | 2.0 ± 0.5 |
|  | Liver (log) (g) | **1.51E-13** | **0.014** | 0.897 | 0.05 ± 0.07 | 0.12 ± 0.07 | 0.01 ± 0.08 | 0.11 ± 0.06 |
|  | Fat pad (log) (g) | **8.13E-12** | 0.252 | 0.199 | -0.25 ± 0.18 | -0.22 ± 0.15 | -0.33 ± 0.20 | -0.06 ± 0.13 |
|  | Heart (log) (g) | **2.39E-08** | **0.017** | 0.938 | -0.77 ± 0.04 | -0.74 ± 0.04 | -0.73 ± 0.04 | -0.68 ± 0.03 |
|  | Kidney (log) (g) | **8.57E-10** | **2.20E-16** | 0.626 | -0.59 ± 0.05 | -0.53 ± 0.05 | -0.62 ± 0.05 | -0.62 ± 0.04 |
|  | Spleen (log) (g) | **4.85E-08** | 0.100 | 0.056 | -0.59 ± 0.05 | -0.53 ± 0.06 | -0.61 ± 0.05 | -0.60 ± 0.04 |
|  | Brown fat (log) | **4.34E-08** | 0.306 | 0.923 | -0.99 ± 0.05 | -0.94 ± 0.04 | -1.03 ± 0.08 | -0.89 ± 0.04 |
|  | Skeletal muscle (log) (g) | **0.018** | 0.616 | 0.960 | -0.49 ± 0.12 | -0.39 ± 0.09 | -0.63 ± 0.12 | -0.38 ± 0.09 |

Figure S1. The multidimensional scaling plot indicates that gene expression libraries clustered by sex (dimension 1) and then by diet (dimension 2). HF = High-fat diet, and LF = Low-fat diet.


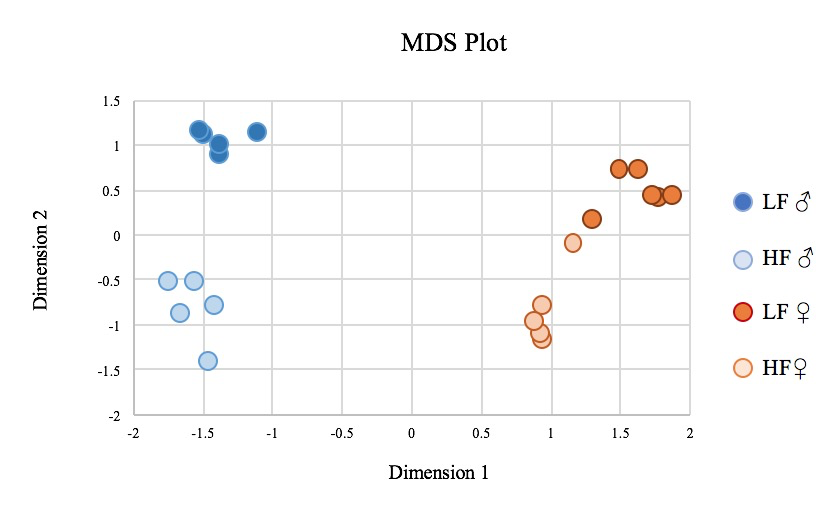


Table S5. Differentially expressed genes due to diet in females (see Excel sheet)

Table S6. Differentially expressed genes due to diet in males (see Excel sheet)

Table S7. The signaling and metabolism pathways up- or downregulated by sex and diet. The ribosome pathway was upregulated in males compared to females, and the cytokine-cytokine and chemokine signaling pathways were upregulated in high-fat mice compared to low-fat mice.

| **Comparison** | **Pathway** | **q-value** | **Up- or**  **Downregulated** |
| --- | --- | --- | --- |
| Males vs. females | mmu03010 Ribosome | 0.012 | up (in males) |
|  | mmu00190 Oxidative phosphorylation | 0.024 | up |
|  | mmu00140 Steroid hormone biosynthesis | 0.052 | down |
|  | mmu00591 Linoleic acid metabolism | 0.052 | down |
|  | mmu00830 Retinol metabolism | 0.052 | down |
| High fat vs. low fat  diet | mmu04060 Cytokine-cytokine receptor interaction | 0.002 | up (in high fat) |
|  | mmu04062 Chemokine signaling pathway | 0.006 | up |
|  | mmu04514 Cell adhesion molecules (CAMs) | 0.016 | up |
|  | mmu04640 Hematopoietic cell lineage | 0.016 | up |
|  | mmu04650 Natural killer cell mediated cytotoxicity | 0.029 | up |
|  | mmu03010 Ribosome | 9.74E-06 | down |
|  | mmu00190 Oxidative phosphorylation | 0.010 | down |

Figure S2. KEGG pathway diagrams, where red indicates upregulation by a high-fat diet and green indicates downregulation. A) The oxidative phosphorylation pathway is significantly downregulated due to a high-fat diet. B) The cytokine-cytokine pathway is significantly upregulated due to a high-fat diet.

A)
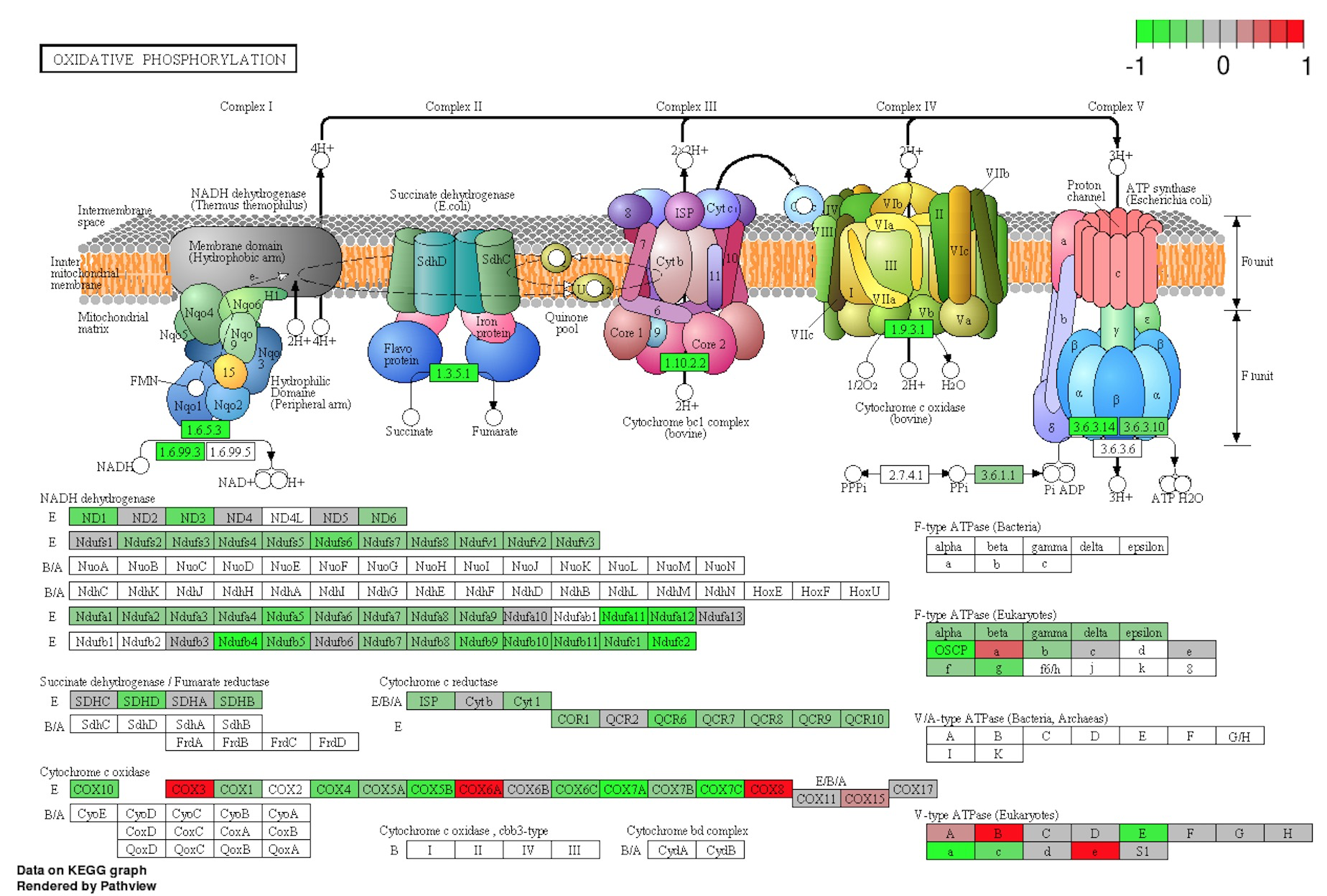


B)
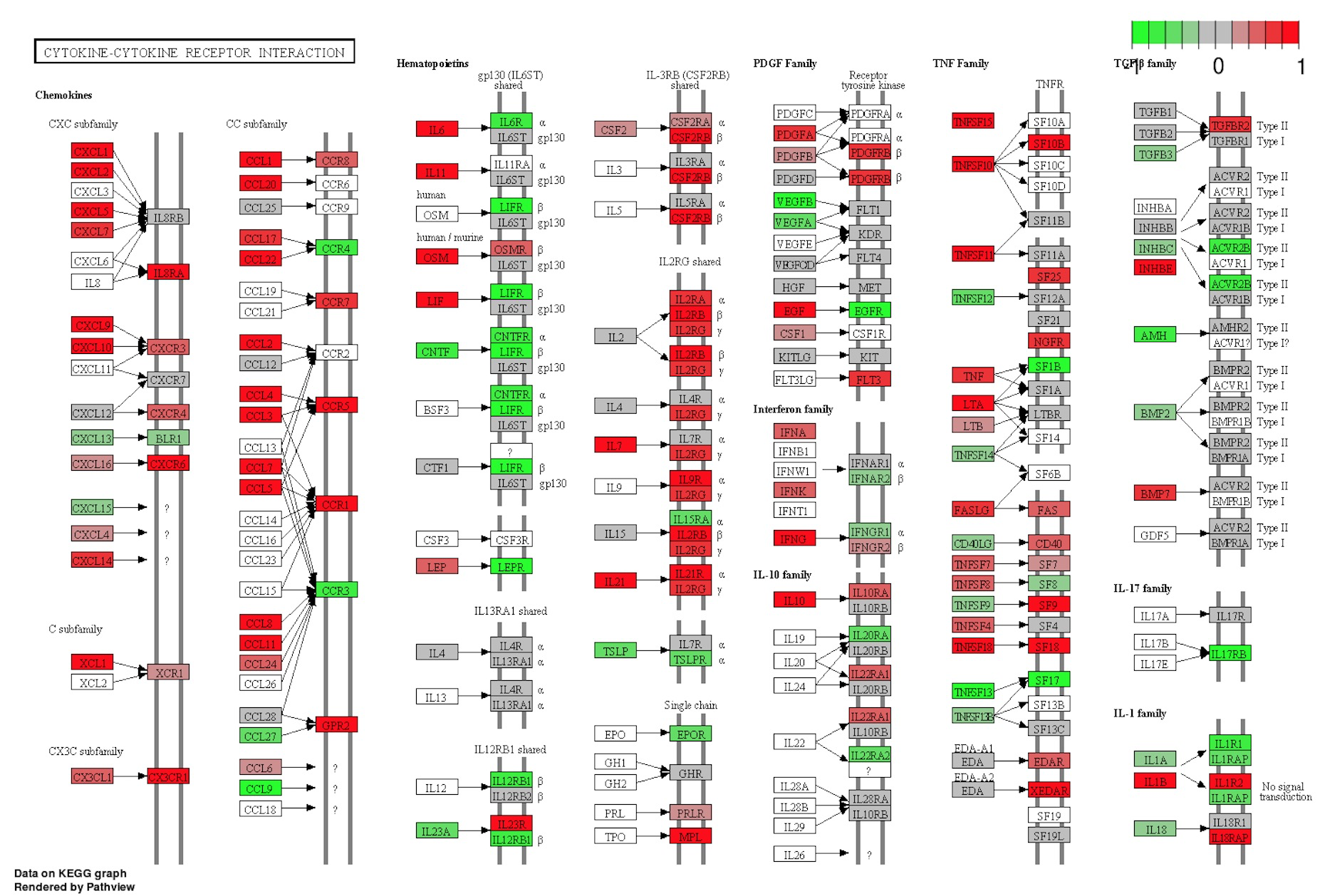


Table S8. The signaling and metabolism pathways up- or downregulated in female mice and male mice due to a high-fat diet. Any genes in these pathways that have a differentially methylated region are listed. Each of the five significant pathways for females was upregulated by a high-fat diet. In males, a high-fat diet upregulated the cytokine-cytokine pathway and downregulated the ribosome and oxidative phosphorylation pathways.

| **Comparison** | **Pathway** | **q-value** | **Up- or**  **Down-regulated** | **Genes in Pathway** | **Genes in the pathway with a DMR** |
| --- | --- | --- | --- | --- | --- |
| High fat vs. low fat females | mmu04060 Cytokine-cytokine receptor interaction | 0.017 | up | 270 | *Acvr1, Bmpr1b, Ccr4, Ccr7, Flt4, Kdr, Lifr, Ngfr, Osm, Pf4, Tnfrsf13b* |
|  | mmu04062 Chemokine signaling pathway | 0.017 | up | 185 | *Adcy1, Adcy5, Adcy7, Ccr4, Ccr7, Dock2, Foxo3, Gng7, Grb2, Hck, Jak3, Mapk3, Nfkb1, Pard3, Pf4, Pik3cd, Pik3r2, Pxn, Stat3, Tiam1, Vav2* |
|  | mmu04514 Cell adhesion molecules (CAMs) | 0.049 | up | 145 | *Cadm1, Cd6, Cdh15, Cldn18, Cldn2, Itga9, Lrrc4b, Mpzl1, Nrxn3, Ntng1, Pecam1, Spn* |
|  | mmu04650 Natural killer cell mediated cytotoxicity | 0.049 | up | 133 | *Cd48, Fyn, Grb2, Lat, Mapk3, Nfatc1, Pik3cd, Pik3r2, Plcg2, Ppp3ca, Sh3bp2, Syk, Vav2, Zap70* |
| High fat vs. low fat  males | mmu04060 Cytokine-cytokine receptor interaction | 0.039 | up | 270 | *Acvr1b, Bmp7, Csf1r, Cx3cr1, Flt3, Osm, Pdgfa, Pdgfra, Tgfbr2* |
|  | mmu03010 Ribosome | 3.3x10^-6^ | down | 154 | NA |
|  | mmu00190 Oxidative phosphorylation | 0.002 | down | 133 | *Atp6v0a1, Lhpp, Ppa1, Ppa2* |

Table S9. Significant GO Biological Processes affected by sex and diet.

| **Comparison** | **GO_ID** | **q-value** | **Up or Down-regulated** |
| --- | --- | --- | --- |
| Sex-by-diet Interaction | GO:0009118 regulation of nucleoside metabolic process | 3.75E-02 | up |
|  | GO:0030811 regulation of nucleotide catabolic process | 3.75E-02 | up |
|  | GO:0033121 regulation of purine nucleotide catabolic process | 3.75E-02 | up |
|  | GO:0046578 regulation of Ras protein signal transduction | 3.75E-02 | up |
|  | GO:0051056 regulation of small GTPase mediated signal transduction | 3.75E-02 | up |
| High-fat vs. low-fat diet | GO:0002684 positive regulation of immune system process | 4.95E-05 | up |
|  | GO:0043207 response to external biotic stimulus | 4.95E-05 | up |
|  | GO:0045087 innate immune response | 4.95E-05 | up |
|  | GO:0051707 response to other organism | 4.95E-05 | up |
|  | GO:0001816 cytokine production | 2.97E-04 | up |
|  | GO:0009607 response to biotic stimulus | 2.97E-04 | up |
|  | GO:0031349 positive regulation of defense response | 2.97E-04 | up |
|  | GO:0050776 regulation of immune response | 2.97E-04 | up |
|  | GO:0009617 response to bacterium | 2.98E-04 | up |
|  | GO:0001817 regulation of cytokine production | 3.85E-04 | up |
|  | GO:0098542 defense response to other organism | 4.12E-04 | up |
|  | GO:0002237 response to molecule of bacterial origin | 6.30E-04 | up |
|  | GO:0060326 cell chemotaxis | 1.78E-03 | up |
|  | GO:0050900 leukocyte migration | 1.88E-03 | up |
|  | GO:0002252 immune effector process | 2.47E-03 | up |
|  | GO:0006935 chemotaxis | 2.47E-03 | up |
|  | GO:0006954 inflammatory response | 2.47E-03 | up |
|  | GO:0031347 regulation of defense response | 2.47E-03 | up |
|  | GO:0032496 response to lipopolysaccharide | 2.47E-03 | up |
|  | GO:0042330 taxis | 2.47E-03 | up |
|  | GO:0045088 regulation of innate immune response | 2.47E-03 | up |
|  | GO:0045321 leukocyte activation | 2.47E-03 | up |
|  | GO:0050663 cytokine secretion | 2.47E-03 | up |
|  | GO:0050778 positive regulation of immune response | 2.47E-03 | up |
|  | GO:0009306 protein secretion | 3.43E-03 | up |
|  | GO:0046649 lymphocyte activation | 3.69E-03 | up |
|  | GO:0030595 leukocyte chemotaxis | 3.80E-03 | up |
|  | GO:0050707 regulation of cytokine secretion | 4.43E-03 | up |
|  | GO:0034341 response to interferon-gamma | 4.53E-03 | up |
|  | GO:0042742 defense response to bacterium | 4.53E-03 | up |
|  | GO:0000280 nuclear division | 4.55E-03 | up |
|  | GO:0007067 mitosis | 4.55E-03 | up |
|  | GO:0042110 T cell activation | 4.55E-03 | up |
|  | GO:0097529 myeloid leukocyte migration | 4.55E-03 | up |
|  | GO:0050729 positive regulation of inflammatory response | 4.89E-03 | up |
|  | GO:0097530 granulocyte migration | 6.56E-03 | up |
|  | GO:0002521 leukocyte differentiation | 6.90E-03 | up |
|  | GO:0002757 immune response-activating signal transduction | 7.14E-03 | up |
|  | GO:0071219 cellular response to molecule of bacterial origin | 8.63E-03 | up |
|  | GO:0001819 positive regulation of cytokine production | 9.12E-03 | up |
|  | GO:0032103 positive regulation of response to external stimulus | 9.97E-03 | up |
|  | GO:0051249 regulation of lymphocyte activation | 1.15E-02 | up |
|  | GO:0045089 positive regulation of innate immune response | 1.18E-02 | up |
|  | GO:0050708 regulation of protein secretion | 1.18E-02 | up |
|  | GO:0071621 granulocyte chemotaxis | 1.31E-02 | up |
|  | GO:0002764 immune response-regulating signaling pathway | 1.37E-02 | up |
|  | GO:0002253 activation of immune response | 1.47E-02 | up |
|  | GO:0071222 cellular response to lipopolysaccharide | 1.47E-02 | up |
|  | GO:0048285 organelle fission | 1.59E-02 | up |
|  | GO:0050865 regulation of cell activation | 1.59E-02 | up |
|  | GO:0071216 cellular response to biotic stimulus | 1.59E-02 | up |
|  | GO:0072676 lymphocyte migration | 1.59E-02 | up |
|  | GO:0002696 positive regulation of leukocyte activation | 1.73E-02 | up |
|  | GO:0030098 lymphocyte differentiation | 1.77E-02 | up |
|  | GO:0050867 positive regulation of cell activation | 1.80E-02 | up |
|  | GO:0030593 neutrophil chemotaxis | 2.07E-02 | up |
|  | GO:0002694 regulation of leukocyte activation | 2.08E-02 | up |
|  | GO:0071345 cellular response to cytokine stimulus | 2.23E-02 | up |
|  | GO:0032101 regulation of response to external stimulus | 2.34E-02 | up |
|  | GO:0050863 regulation of T cell activation | 2.34E-02 | up |
|  | GO:1990266 neutrophil migration | 2.41E-02 | up |
|  | GO:0071346 cellular response to interferon-gamma | 2.63E-02 | up |
|  | GO:0050715 positive regulation of cytokine secretion | 2.82E-02 | up |
|  | GO:0051251 positive regulation of lymphocyte activation | 2.82E-02 | up |
|  | GO:0002685 regulation of leukocyte migration | 4.36E-02 | up |
|  | GO:0032655 regulation of interleukin-12 production | 4.36E-02 | up |
|  | GO:0050870 positive regulation of T cell activation | 4.63E-02 | up |
|  | GO:0043900 regulation of multi-organism process | 4.64E-02 | up |
|  | GO:0034097 response to cytokine | 5.18E-02 | up |
|  | GO:0002443 leukocyte mediated immunity | 5.27E-02 | up |
|  | GO:0030217 T cell differentiation | 5.27E-02 | up |
|  | GO:0002687 positive regulation of leukocyte migration | 5.31E-02 | up |
|  | GO:0032615 interleukin-12 production | 5.45E-02 | up |
| High fat vs. low fat females | GO:0043207 response to external biotic stimulus | 1.27E-03 | up |
|  | GO:0045087 innate immune response | 1.27E-03 | up |
|  | GO:0051707 response to other organism | 1.27E-03 | up |
|  | GO:0002684 positive regulation of immune system process | 2.29E-03 | up |
|  | GO:0098542 defense response to other organism | 3.95E-03 | up |
|  | GO:0009607 response to biotic stimulus | 6.07E-03 | up |
|  | GO:0002252 immune effector process | 6.10E-03 | up |
|  | GO:0009617 response to bacterium | 6.10E-03 | up |
|  | GO:0050776 regulation of immune response | 6.52E-03 | up |
|  | GO:0001816 cytokine production | 7.54E-03 | up |
|  | GO:0001817 regulation of cytokine production | 1.22E-02 | up |
|  | GO:0002237 response to molecule of bacterial origin | 1.22E-02 | up |
|  | GO:0031349 positive regulation of defense response | 1.22E-02 | up |
|  | GO:0060326 cell chemotaxis | 1.35E-02 | up |
|  | GO:0042742 defense response to bacterium | 1.79E-02 | up |
|  | GO:0050900 leukocyte migration | 1.79E-02 | up |
|  | GO:0009306 protein secretion | 1.85E-02 | up |
|  | GO:0050663 cytokine secretion | 1.90E-02 | up |
|  | GO:0030595 leukocyte chemotaxis | 2.11E-02 | up |
|  | GO:0006954 inflammatory response | 2.35E-02 | up |
|  | GO:0034341 response to interferon-gamma | 2.44E-02 | up |
|  | GO:0050778 positive regulation of immune response | 2.58E-02 | up |
|  | GO:0032496 response to lipopolysaccharide | 3.46E-02 | up |
|  | GO:0050729 positive regulation of inflammatory response | 3.97E-02 | up |
|  | GO:0031347 regulation of defense response | 4.36E-02 | up |
|  | GO:0045088 regulation of innate immune response | 4.36E-02 | up |
|  | GO:0050707 regulation of cytokine secretion | 4.36E-02 | up |
|  | GO:0097529 myeloid leukocyte migration | 4.36E-02 | up |
|  | GO:0097530 granulocyte migration | 4.36E-02 | up |
| High-fat vs. low-fat diet males | GO:0002684 positive regulation of immune system process | 2.03E-03 | up |
|  | GO:0045087 innate immune response | 3.38E-03 | up |
|  | GO:0043207 response to external biotic stimulus | 4.53E-03 | up |
|  | GO:0051707 response to other organism | 4.53E-03 | up |
|  | GO:0001816 cytokine production | 5.86E-03 | up |
|  | GO:0031349 positive regulation of defense response | 5.86E-03 | up |
|  | GO:0001817 regulation of cytokine production | 6.70E-03 | up |
|  | GO:0009617 response to bacterium | 6.70E-03 | up |
|  | GO:0002237 response to molecule of bacterial origin | 6.81E-03 | up |
|  | GO:0006935 chemotaxis | 6.81E-03 | up |
|  | GO:0009607 response to biotic stimulus | 6.81E-03 | up |
|  | GO:0042330 taxis | 6.81E-03 | up |
|  | GO:0050776 regulation of immune response | 6.81E-03 | up |
|  | GO:0045321 leukocyte activation | 1.15E-02 | up |
|  | GO:0007067 mitosis | 1.20E-02 | up |
|  | GO:0046649 lymphocyte activation | 1.23E-02 | up |
|  | GO:0042110 T cell activation | 1.55E-02 | up |
|  | GO:0032496 response to lipopolysaccharide | 1.64E-02 | up |
|  | GO:0098542 defense response to other organism | 1.64E-02 | up |
|  | GO:0031347 regulation of defense response | 1.74E-02 | up |
|  | GO:0045088 regulation of innate immune response | 1.74E-02 | up |
|  | GO:0050663 cytokine secretion | 1.74E-02 | up |
|  | GO:0050900 leukocyte migration | 1.74E-02 | up |
|  | GO:0002521 leukocyte differentiation | 2.09E-02 | up |
|  | GO:0050707 regulation of cytokine secretion | 2.09E-02 | up |
|  | GO:0060326 cell chemotaxis | 2.19E-02 | up |
|  | GO:0050778 positive regulation of immune response | 2.27E-02 | up |
|  | GO:0034341 response to interferon-gamma | 2.40E-02 | up |
|  | GO:0006954 inflammatory response | 2.44E-02 | up |
|  | GO:0071345 cellular response to cytokine stimulus | 2.44E-02 | up |
|  | GO:0000280 nuclear division | 2.49E-02 | up |
|  | GO:0002757 immune response-activating signal transduction | 2.81E-02 | up |
|  | GO:0030098 lymphocyte differentiation | 2.81E-02 | up |
|  | GO:0051056 regulation of small GTPase mediated signal transduction | 3.30E-02 | up |
|  | GO:0051249 regulation of lymphocyte activation | 3.30E-02 | up |
|  | GO:0030334 regulation of cell migration | 3.34E-02 | up |
|  | GO:0071219 cellular response to molecule of bacterial origin | 3.34E-02 | up |
|  | GO:0001819 positive regulation of cytokine production | 3.37E-02 | up |
|  | GO:0050865 regulation of cell activation | 3.37E-02 | up |
|  | GO:0097529 myeloid leukocyte migration | 3.37E-02 | up |
|  | GO:0045089 positive regulation of innate immune response | 3.52E-02 | up |
|  | GO:0050867 positive regulation of cell activation | 3.97E-02 | up |
|  | GO:0030595 leukocyte chemotaxis | 4.24E-02 | up |
|  | GO:0046578 regulation of Ras protein signal transduction | 4.36E-02 | up |
|  | GO:0071216 cellular response to biotic stimulus | 4.36E-02 | up |
|  | GO:0032103 positive regulation of response to external stimulus | 4.51E-02 | up |
|  | GO:0032880 regulation of protein localization | 4.51E-02 | up |
|  | GO:0050729 positive regulation of inflammatory response | 4.51E-02 | up |
|  | GO:0071222 cellular response to lipopolysaccharide | 4.51E-02 | up |
|  | GO:0072676 lymphocyte migration | 4.51E-02 | up |
|  | GO:2000145 regulation of cell motility | 4.51E-02 | up |
|  | GO:0097530 granulocyte migration | 4.52E-02 | up |
|  | GO:0002694 regulation of leukocyte activation | 4.56E-02 | up |
|  | GO:0002696 positive regulation of leukocyte activation | 4.56E-02 | up |
|  | GO:0009306 protein secretion | 4.56E-02 | up |
|  | GO:0002764 immune response-regulating signaling pathway | 4.60E-02 | up |
|  | GO:0042742 defense response to bacterium | 5.01E-02 | up |
|  | GO:0034097 response to cytokine | 5.09E-02 | up |
|  | GO:0002253 activation of immune response | 5.24E-02 | up |
|  | GO:0030097 hemopoiesis | 5.24E-02 | up |
|  | GO:0032101 regulation of response to external stimulus | 5.24E-02 | up |

Table S10. Differentially methylated regions due to diet in females (see Excel sheet).

Table S11. Differentially methylated regions due to diet in males (see Excel sheet).

Table S12. The number of differentially methylated regions (DMRs) due to diet and sex at three different q-value cutoffs. There were thousands of methylation differences due to diet, and even more due to sex. A greater proportion of the DMRs fell on the X-chromosome when comparing across sexes than when comparing across diet treatments. HF = High-fat diet, and LF = Low-fat diet.

| **Comparison** | **Group** | **<0.05** | **<0.01** | **<0.001** | **DMRs in X (%)** |
| --- | --- | --- | --- | --- | --- |
| Different diets | LF ♀ vs. HF ♀ | 38,865 | 2,356 | 375 | 100 (0.3%) |
|  | LF ♂ vs. HF ♂ | 31,549 | 1,539 | 314 | 17 (0.1%) |
| Different sexes | HF ♀ vs. HF ♂ | 36,876 | 3,831 | 1,250 | 1,045 (2.8%) |
|  | LF ♀ vs. LF ♂ | 44,076 | 5,632 | 1,716 | 994 (2.3%) |

Table S13. The number of genes in the mouse liver with: at least one diet-induced differentially methylated region (DMR) within the gene body, more than one DMR in the gene body, and at least one DMR in the promoter, defined as within 2 kb upstream of the transcription start site (q < 0.05).

|  | **Genes with ≥ 1 DMR in gene body** | **Genes with > 1 DMR in gene body** | **Genes with ≥ 1 DMR in promoter** |
| --- | --- | --- | --- |
| **Females** | 7,814 (38.3%) | 3,912 (19.2%) | 2,146 (10.5%) |
| **Males** | 7,086 (34.7%) | 3,375 (16.5%) | 1,548 (7.6%) |

Figure S3. A) Venn Diagram illustrating the number of genes whose expression was found to be altered by a high-fat diet in our study of SM/J mice, compared to those found by other researchers using C57BL/6 mice. The present study replicated between 28-40% of the genes found in other studies. B) Only two genes were found by all three studies that used C57BL/6 mice, illustrating the difficulty of replication in studies of how dietary fat alters gene expression.

A)
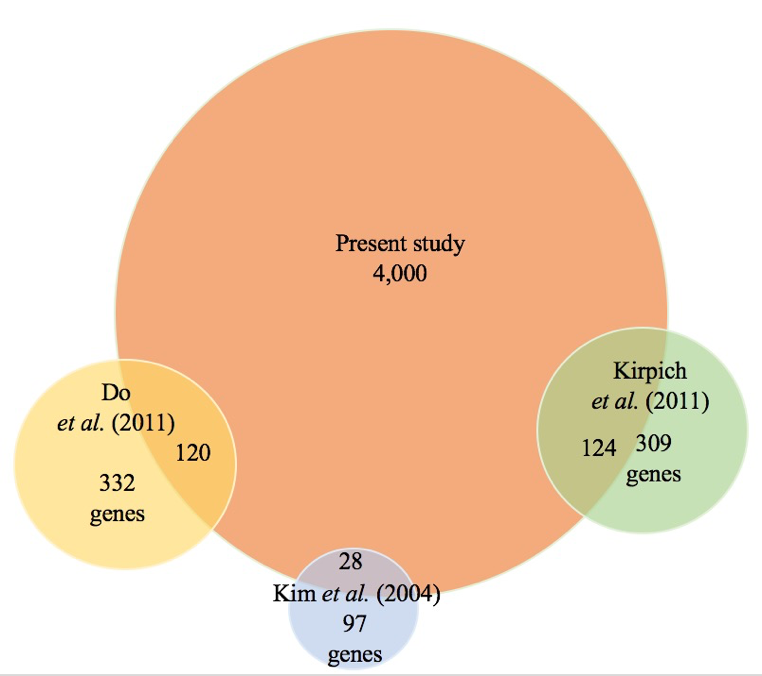
 B)
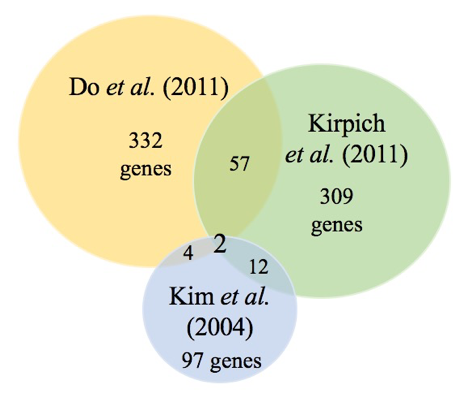


Table S14. Comparison of SM/J expression levels from the present study with expression levels altered by a high-fat diet in the nine strains studied by Shockley et al. (37) (See Excel sheet).
